# Thiocarbonyl-enabled ferrocene C–H nitrogenation by cobalt(III) catalysis: thermal and mechanochemical

**DOI:** 10.3762/bjoc.14.131

**Published:** 2018-06-25

**Authors:** Santhivardhana Reddy Yetra, Zhigao Shen, Hui Wang, Lutz Ackermann

**Affiliations:** 1 Institut für Organische und Biomolekulare Chemie, Georg-August-Universität Göttingen, Tammannstraße 2, 37077 Göttingen, Germany

**Keywords:** amidation, C–H activation, cobalt, ferrocene, mechanochemistry

## Abstract

Versatile C–H amidations of synthetically useful ferrocenes were accomplished by weakly-coordinating thiocarbonyl-assisted cobalt catalysis. Thus, carboxylates enabled ferrocene C–H nitrogenations with dioxazolones, featuring ample substrate scope and robust functional group tolerance. Mechanistic studies provided strong support for a facile organometallic C–H activation manifold.

## Introduction

**Figure 1:**
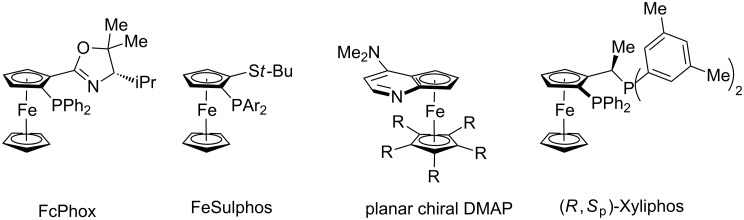
Selected ferrocene-based ligands and organocatalysts.

C–H activation has surfaced as a transformative tool in molecular sciences [1–9]. While major advances have been accomplished with precious 4d transition metals, recent focus has shifted towards more sustainable base metals [10–17], with considerable progress by earth-abundant cobalt catalysts [18–22]. In this context, well-defined cyclopentadienyl-derived cobalt(III) complexes have proven instrumental for enabling a wealth of C–H transformations [23–41], prominently featuring transformative C–H nitrogenations [42–43] in an atom- and step-economical fashion [44–59]. Within our program on cobalt-catalyzed C–H activation [60–68], we have now devised C–H nitrogenations assisted by weakly-coordinating [69] thiocarbonyls [70–71], allowing the direct C–H activation on substituted ferrocenes [72–93] – key structural motifs of powerful transition metal catalyst ligands and organocatalysts (Figure 1) [94–97]. During the preparation of this article, the use of strongly-coordinating, difficult to remove directing groups has been reported [70–71]. In sharp contrast, notable features of our approach include (i) cobalt-catalyzed C–H amidations of thiocarbonylferrocenes by weak coordination, (ii) thermal and mechanochemical [98–100] cobalt-catalyzed ferrocene C–H nitrogenations, (iii) versatile access to synthetically useful aminoketones, and (iv) key mechanistic insights on facile C–H cobaltation.

## Results and Discussion

We initiated our studies by probing various reaction conditions for the envisioned C–H amidation of ferrocene **1a** (Table 1). Among a variety of ligands, N-heterocyclic carbenes and phosphines provided unsatisfactory results (Table 1, entries 1–3), while the product **3aa** was formed when using amino acid derivatives, albeit as of yet in a racemic fashion (Table 1, entries 4–7). Yet, optimal catalytic performance was realized with 1-AdCO_2_H (Table 1, entries 8 and 9) [101–104], particularly when using DCE as the solvent (Table 1, entries 9–12). A control experiment verified the essential nature of the cobalt catalyst (Table 1, entry 13). In contrast to the thiocarbonyl-assisted C–H amidation, the corresponding ketone failed thus far to deliver the desired product, under otherwise identical reaction conditions.

**Table 1:** Thiocarbonyl-assisted C−H nitrogenation of ferrocene **1a**.^a^

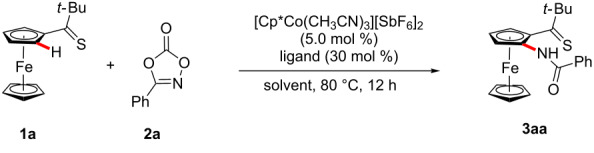			

Entry	Solvent	Ligand	Yield (%)

1	DCE	–	–
2	DCE	IMes·HCl	–
3	DCE	PPh_3_	–
4	DCE	Boc-Leu-OH	40
5	DCE	Boc-Val-OH	55
6	DCE	Boc-Pro-OH	30
7	DCE	Boc-Ala-OH	62
8	DCE	MesCO_2_H	80
**9**	**DCE**	**1-AdCO_2_H**	**84**
10	1,4-dioxane	1-AdCO_2_H	75
11	toluene	1-AdCO_2_H	79
12	GVL	1-AdCO_2_H	35
13	DCE	1-AdCO_2_H	–^b^

^a^Reaction conditions: **1a** (0.13 mmol), **2a** (0.15 mmol), ligand (30 mol %), [Co] (5.0 mol %), solvent (1.0 mL). ^b^Reaction performed in the absence of [Cp*Co(CH_3_CN)_3_][SbF_6_]_2_. Yields of isolated product.

**Scheme 1:**
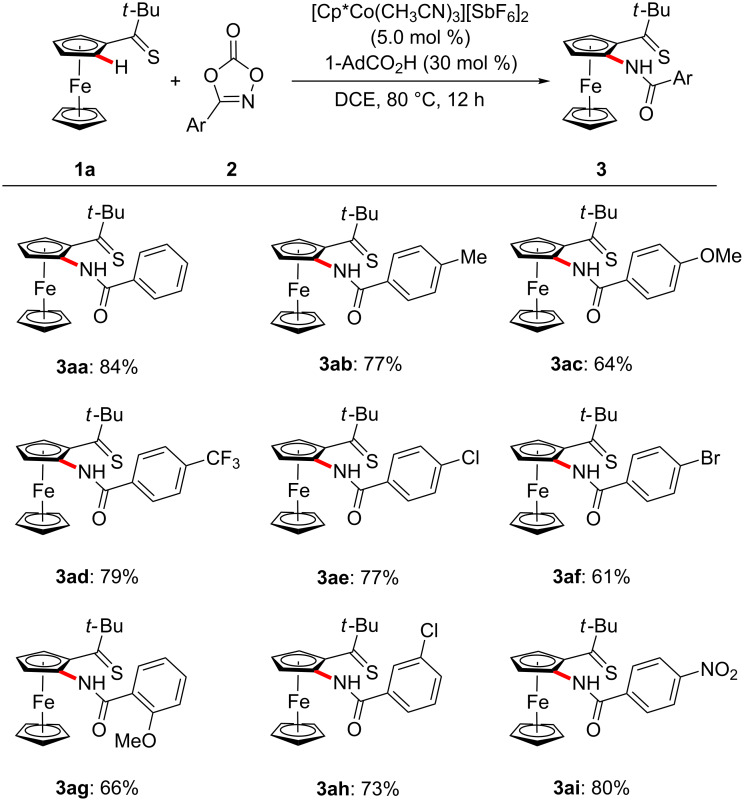
Scope of substituted dioxazolones **2**.

With the optimized reaction conditions in hand, we explored the robustness of the cobalt-catalyzed ferrocene C–H amidation with a variety of 1,4,2-dioxazol-5-ones **2** (Scheme 1). Hence, the chemoselectivity of the cobalt catalyst was reflected by fully tolerating sensitive electrophilic functional groups, including amido, chloro, bromo and nitro substituents in the *para*-, *meta*- and even the more congested *ortho*-position.

**Scheme 2:**
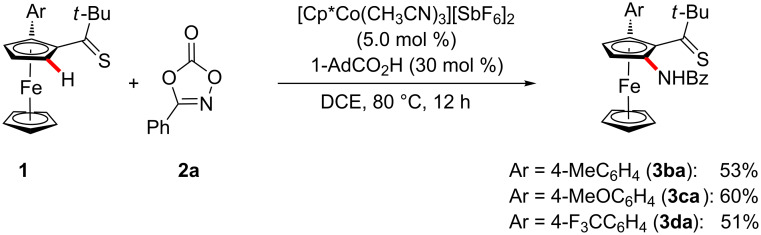
C–H Amidation of arylated ferrocenes **1**.

The versatile cobalt-catalyzed C–H amidation was not limited to mono-substituted ferrocenes **1** (Scheme 2). Indeed, the arylated ferrocenes **1b**–**d** were identified as viable substrates likewise.

**Scheme 3:**
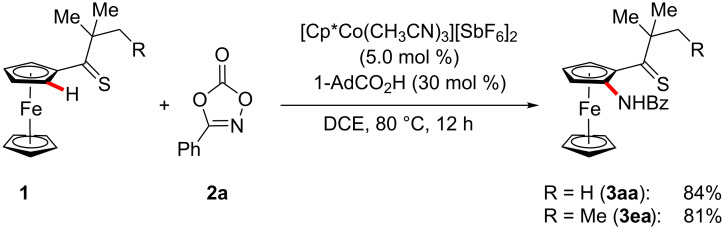
Thiocarbonyl-assisted C–H amidation.

Moreover, differently substituted thiocarbonyls **1** were found to be amenable within the cobalt-catalyzed C–H amidation manifold by weak-coordination (Scheme 3).

**Scheme 4:**
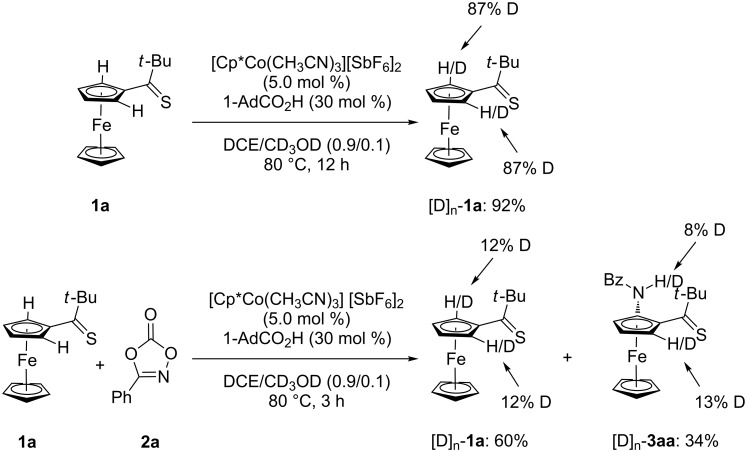
H/D Exchange reactions.

Given the versatility of the cobalt-catalyzed C–H nitrogenation, we became intrigued to delineating its mode of action. To this end, C–H amidations in the presence of isotopically labelled co-solvents led to a significant H/D scrambling in proximity to the thiocarbonyl group. These findings are indicative of a reversible, thus facile organometallic C–H cobaltation regime (Scheme 4).

**Scheme 5:**
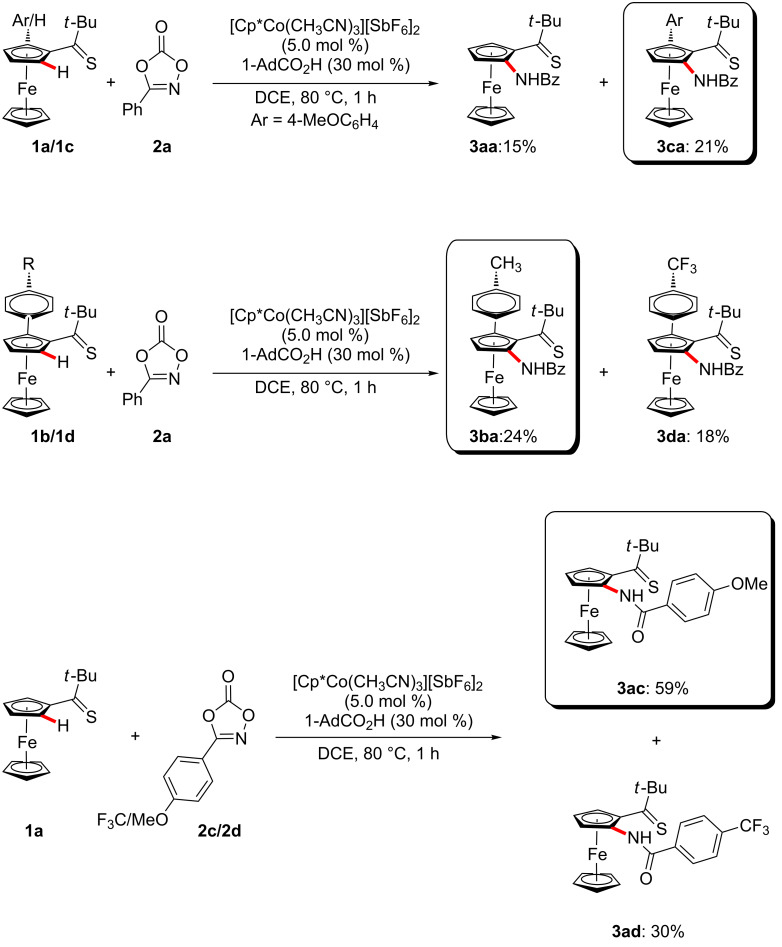
Intermolecular competition experiments.

Next, intermolecular competition experiments revealed that electron-rich arylated thiocarbonylferrocene **1** reacted preferentially, which can be rationalized with a base-assisted internal electrophilic substitution (BIES) [24,105] C–H cobaltation mechanism. In addition, the electron-rich amidating reagent **2c** was found to be inherently more reactive (Scheme 5).

**Scheme 6:**
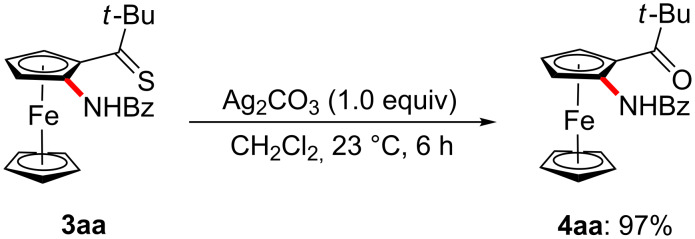
Synthesis of aminoketone **4aa**.

As to further late-stage manipulation of the thus-obtained products, the amidated thiocarbonylferrocene **3aa** could be easily transformed into the corresponding synthetically useful aminoketone **4aa** (Scheme 6), illustrating the unique synthetic utility of our strategy.

**Scheme 7:**
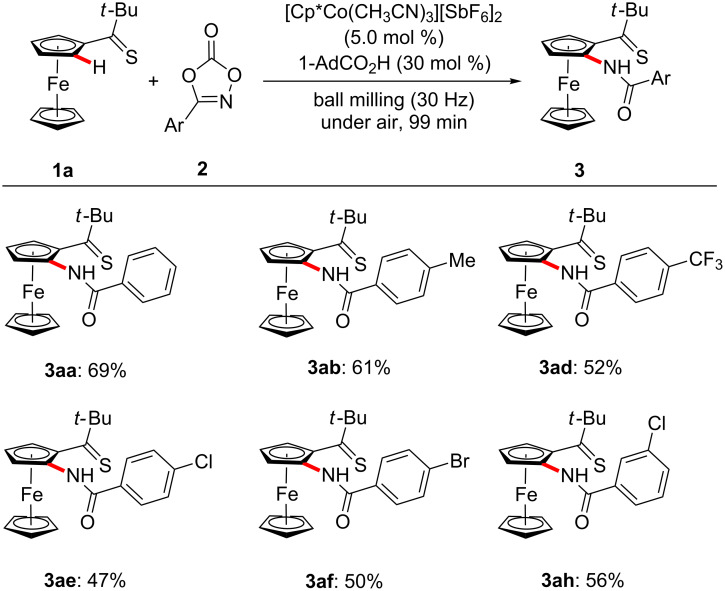
Mechanochemical ferrocene C–H nitrogenation.

Mechanochemical molecular synthesis has attracted recent renewed attention as an attractive alternative for facilitating sustainable organic syntheses [106]. Thus, we were delighted to observe that the mechanochemical C–H nitrogenations proved likewise viable by thiocarbonyl assistance in an effective manner (Scheme 7).

## Conclusion

In conclusion, we have reported on the unprecedented cobalt-catalyzed C–H nitrogenation of ferrocenes by weakly-coordinating thiocarbonyls. The carboxylate-assisted cobalt catalysis was characterized by high functional group tolerance and ample substrate scope. Mechanistic studies provided evidence for a facile C–H activation. The C–H amidation was achieved in a thermal fashion as well as by means of mechanochemistry, providing access to synthetically meaningful aminoketones.

## Supporting Information

File 1 Experimental procedures, characterization data, and NMR spectra for new compounds.
