# Supplementary material for: Thiocarbonyl-enabled ferrocene C–H nitrogenation by cobalt(III) catalysis: thermal and mechanochemical
Source: Beilstein J Org Chem. 2018 Jun 25;14:1546–53. doi: 10.3762/bjoc.14.131 (PMC6037017; doi:10.3762/bjoc.14.131)
Supplement: File 1 — Experimental procedures, characterization data, and NMR spectra for new compounds. [file Beilstein_J_Org_Chem-14-1546-s001.pdf]

Supporting Information

for

Thiocarbonyl-enabled ferrocene C–H nitrogenation by cobalt(III) catalysis: thermal and mechanochemical

Santhivardhana Reddy Yetra[‡], Zhigao Shen[‡], Hui Wang and Lutz Ackermann*[§]

Address: Institut für Organische und Biomolekulare Chemie, Georg-August-Universität Göttingen, Tammannstraße 2, 37077 Göttingen, Germany

*Corresponding author

Email: Lutz Ackermann - Lutz.Ackermann@chemie.uni-goettingen.de

[§]http://www.org.chemie.uni-goettingen.de/ackermann/

[‡]Equal contributors

Experimental procedures, characterization data, and NMR spectra for new compounds

Table of content

General remarks **S2**

Table S1: Optimization studies for C−H nitrogenation of ferrocene **S3**

General procedure A: C−H nitrogenation of ferrocene with dioxazolones **S4**

General procedure B: mechanochemical C−H nitrogenation of ferrocene with dioxazolones **S4**

Characterization data for ferrocene amides 3 .... **S5**

H/D Exchange experiments **S12**

Intermolecular competition experiments **S14**

Late-stage diversification **S16**

References **S17**

^{1}H, ^{13}C and ^{19}F NMR spectra **S18**

General remarks

All catalytic reactions were carried out under inert atmosphere using pre-dried 25 mL pressure tubes. Toluene was purified by an MBraun MB SPS-800 solvent purification system. DCE was distilled over CaH_2 prior to use. The following starting materials were synthesized according to previously described methods: Substituted ferrocenes **1a**–**e**,[1] dioxazolones **2a**–**g**,[2] $[Cp^*Co(CH_3CN)_3](SbF_6)_2$.[3] Other chemicals were obtained from commercial sources and used without further purification. Yields refer to isolated compounds, estimated to be >95% pure as determined by ^{1}H NMR and GC analysis. TLC: Merck, TLC Silica gel 60 F254. Chromatographic separations were carried out on Merck Silica 60 (0.040–0.063 mm). All IR spectra were recorded on a Bruker ATR FT-IR Alpha device. In situ IR spectra were acquired using Mettler-Toledo iC IR software version 7.0.297 in the range of 650–2200 cm^{-1} with 4 cm^{-1} resolution. MS: EIMS: Finnigan MAT 95, 70 eV; ESIMS: Finnigan LCQ. High-resolution mass spectrometry (HRMS): APEX IV 7T FTICR, Bruker Daltonic. Melting points (M.p.): Büchi 540 capillary melting point apparatus, values are uncorrected. NMR spectra were recorded on Varian Mercury VX 300, Inova-500, Inova-600 and Bruker Avance 300, Avance III 300, Avance III HD 400, Avance III 400, Avance III HD 500 instruments, if not otherwise specified, chemical shifts (δ) are provided in ppm. Ball mill: Retsch MM400.

Table S1: Optimization studies for C−H nitrogenation of ferrocene

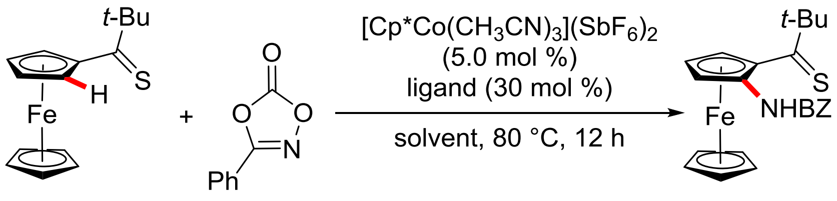

Entry	Solvent	Ligand	Yield (%)[a]
1	DCE	---	---
2	DCE	IMes·HCl	---
3	DCE	PPh_3	---
4	DCE	Boc-Leu-OH	40
5	DCE	Boc-Val-OH	55
6	DCE	Boc-Pro-OH	30
7	DCE	Boc-Ala-OH	62
8	DCE	$MesCO_2H$	80
**9**	**DCE**	**1-AdCO$_2$H**	**84**
10	1,4-dioxane	1-AdCO$_2$H	75
11	toluene	1-AdCO$_2$H	79
12	GVL	1-AdCO$_2$H	35
13	DCE	1-AdCO$_2$H	---[b]

[a]Reaction conditions: **1a** (0.13 mmol), **2a** (0.15 mmol), ligand (30 mol %), [Co] (5.0 mol %), solvent (1.0 mL). [b]Reaction performed without [Cp*Co(CH_3CN)$_3$](SbF_6)$_2$. Yields of isolated product.

General procedure A: C−H nitrogenation of ferrocene with dioxazolones

Thiocarbonylferrocene **1** (0.25 mmol, 1.0 equiv), dioxazolone **2** (0.3 mmol, 1.2 equiv), 1-AdCO_2H (13.5 mg, 30 mol %) and [Cp*Co(CH_3CN)$_3$](SbF_6)$_2$ (**4**) (6.9 mg, 5.0 mol %) in DCE (1.0 mL) were stirred at 80 °C for 12 h under nitrogen atmosphere. At ambient temperature, the mixture was transferred into a round bottom flask with CH_2Cl_2 (15 mL) and concentrated in vacuo. Purification by column chromatography on silica gel afforded the desired products **3**.

General procedure B: mechanochemial C−H nitrogenation of ferrocene with dioxazolones

Thiocarbonylferrocene **1** (0.25 mmol, 1.0 equiv), dioxazolone **2** (0.3 mmol, 1.2 equiv), 1-AdCO_2H (13.5 mg, 30 mol %) and [Cp*Co(CH_3CN)$_3$](SbF_6)$_2$ (**4**) (6.9 mg, 5.0 mol %) were milled in a 10 mL tungsten carbide milling jar with one milling ball made of the same material (10 mm in diameter). After 90 min of reaction at 30 Hz, the milling was stopped and the mixture was transferred into a round bottom flask with CH_2Cl_2 (20 mL) and concentrated in vacuo. Purification by column chromatography on silica gel afforded the desired products **3**.

Characterization data for ferrocene amides 3

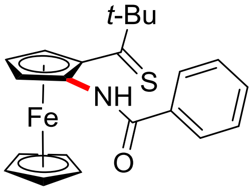

***N*-[2-(2,2-Dimethylpropanethioyl)ferrocenyl]benzamide** (**3aa**)

The general procedure **A** was followed using 2,2-dimethyl-1-ferrocenylpropane-1-thione (**1a**) (71 mg, 0.25 mmol) and 3-phenyl-1,4,2-dioxazol-5-one (**2a**) (49 mg, 0.3 mmol). Purification by column chromatography (*n*-hexane/EtOAc: 19/1) yielded **3aa** (85 mg, 84%) as a blue foam. **M. p.** = 100–102 °C. **^{1}H NMR** (400 MHz, $CDCl_3$): δ = 11.66 (brs, 1H), 8.03–7.98 (m, 2H), 7.57–7.48 (m, 3H), 6.41 (dd, J = 3.0, 1.5 Hz, 1H), 4.81 (dd, J = 3.0, 1.5 Hz, 1H), 4.65 (td, J = 3.0, 0.5 Hz, 1H), 4.09 (s, 5H), 1.54 (s, 9H). **^{13}C NMR** (100 MHz, $CDCl_3$): δ = 261.9 (C_q), 166.3 (C_q), 134.8 (C_q), 131.8 (CH), 128.8 (CH), 127.5 (CH), 102.5 (C_q), 76.1 (C_q), 73.3 (CH), 68.7 (CH), 68.4 (CH), 66.4 (CH), 52.9 (C_q), 32.4 (CH_3). **IR** (ATR): 2958, 2922, 1668, 1525, 1485, 1313, 700 cm^{-1}. **MS** (ESI) *m/z* (relative intensity): 428 (90) $[M+Na]^+$, 406 (100) $[M+H]^+$, 381 (20). **HR-MS** (ESI) *m/z* calcd for $C_{22}H_{24}FeNOS$ $[M+H]^+$ 406.0923, found 406.0917.

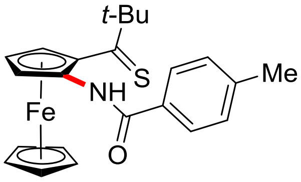

***N*-[2-(2,2-Dimethylpropanethioyl)ferrocenyl]-4-methylbenzamide** (**3ab**)

The general procedure **A** was followed using 2,2-dimethyl-1-ferrocenylpropane-1-thione (**1a**) (71 mg, 0.25 mmol) and 3-(4-methylphenyl)-1,4,2-dioxazol-5-one (**2b**) (53 mg, 0.3 mmol). Purification by column chromatography (*n*-hexane/EtOAc: 10/1) yielded **3ab** (83 mg, 77%) as a blue foam. **M. p.** = 106–108 °C. **^{1}H NMR** (400 MHz, $CDCl_3$): δ = 11.58 (s, 1H), 7.87 (d, J = 8.0 Hz, 2H), 7.29 (d, J = 8.0 Hz, 2H), 6.39 (dd, J = 3.0, 1.5 Hz, 1H), 4.78 (dd, J = 3.0, 1.5 Hz, 1H), 4.63 (dd, J = 3.0, 3.0 Hz, 1H), 4.06 (s, 5H), 2.41 (s, 3H), 1.52 (s, 9H). **^{13}C NMR** (100 MHz, $CDCl_3$): δ = 261.7 (C_q), 166.2 (C_q), 142.1 (C_q), 131.9 (C_q), 129.4 (CH), 127.4 (CH), 102.5 (C_q), 75.9 (C_q), 73.1 (CH), 68.5 (CH), 68.2 (CH), 66.1 (CH), 52.7 (C_q), 32.3 (CH_3), 21.5 (CH_3). **IR** (ATR): 3048, 1611, 1491, 805, 532 cm^{-1}. **MS** (ESI) *m/z* (relative

intensity): 442 (100) $[M+Na]^+$, 420 (20) $[M+H]^+$. **HR-MS** (ESI) *m/z* calcd for $C_{23}H_{26}FeNO_2S$ $[M+H]^+$ 420.1087, found 420.1090.

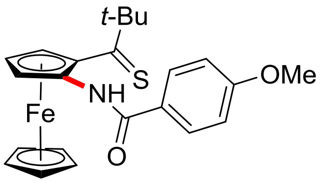

***N*-[2-(2,2-Dimethylpropanethioyl)ferrocenyl]-4-methoxybenzamide** (**3ac**)

The general procedure **A** was followed using 2,2-dimethyl-1-ferrocenylpropane-1-thione (**1a**) (71 mg, 0.25 mmol) and 3-(4-methoxyphenyl)-1,4,2-dioxazol-5-one (**2c**) (58 mg, 0.3 mmol). Purification by column chromatography (*n*-hexane/EtOAc: 10/1) yielded **3ac** (70 mg, 64%) as a blue foam. **M. p.** = 105–106 °C. **^{1}H NMR** (300 MHz, $CDCl_3$): δ = 11.57 (brs, 1H), 7.97 (d, J = 8.7 Hz, 2H), 7.01 (d, J = 8.7 Hz, 2H), 6.40 (dd, J = 2.7, 1.4 Hz, 1H), 4.81 (dd, J = 2.7, 1.4 Hz, 1H), 4.65 (t, J = 2.7 Hz, 1H), 4.09 (s, 5H), 3.89 (s, 3H), 1.54 (s, 9H). **^{13}C NMR** (75 MHz, $CDCl_3$) δ = 261.8 (C_q), 165.8 (C_q), 162.4 (C_q), 129.3 (CH), 127.1 (C_q), 113.9 (CH), 102.8 (C_q), 75.9 (C_q), 73.2 (CH), 68.6 (CH), 68.2 (CH), 66.1 (CH), 55.5 (CH_3), 52.8 (C_q), 32.3 (CH_3). **IR** (ATR): 3047, 1607, 1501, 1240, 1026, 805, 487 cm^{-1}. **MS** (ESI) *m/z* (relative intensity): 474 (100), 458 (60) $[M+Na]^+$, 436 (60) $[M+H]^+$, 174 (20). **HR-MS** (ESI) *m/z* calcd for $C_{23}H_{26}FeNO_2S$ $[M+H]^+$ 436.1029, found 436.1028.

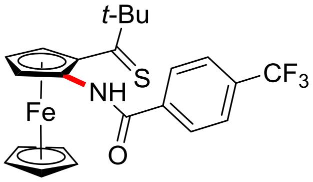

***N*-[2-(2,2-Dimethylpropanethioyl)ferrocenyl]-4-(trifluoromethyl)benzamide (3ad)**

The general procedure **A** was followed using 2,2-dimethyl-1-ferrocenylpropane-1-thione (**1a**) (71 mg, 0.25 mmol) and 3-[4-(trifluoromethyl)phenyl]-1,4,2-dioxazol-5-one (**2d**) (69 mg, 0.3 mmol). Purification by column chromatography (*n*-hexane/EtOAc: 10/1) yielded **3ad** (88 mg, 79%) as blue foam. **M. p.** = 92–93 °C. **^{1}H NMR** (300 MHz, $CDCl_3$): δ = 11.86 (s, 1H), 8.13 (d, J = 8.1 Hz, 2H), 7.80 (d, J = 8.1 Hz, 2H), 6.42 (dd, J = 3.0, 1.5 Hz, 1H), 4.87 (dd, J = 3.0, 1.5 Hz, 1H), 4.71 (t, J = 3.0 Hz, 1H), 4.12 (s, 5H), 1.56 (s, 9H). **^{13}C NMR** (75 MHz, $CDCl_3$): δ = 262.2 (C_q), 164.9 (C_q), 138.1 (C_q), 133.4 (q, $^2J_{C-F}$ = 32.7 Hz, C_q), 128.0 (CH), 125.9 (q, $^3J_{C-F}$ = 3.7 Hz, CH), 123.8 (q, $^1J_{C-F}$ = 272.6 Hz, C_q), 101.9 (C_q), 76.0 (C_q), 73.4 (CH), 68.9 (CH), 68.5 (CH), 66.8 (CH), 53.0 (C_q), 32.5 (CH_3). **^{19}F NMR** (282 MHz, $CDCl_3$) δ = −62.89. **IR** (ATR): 3029, 1616, 1559, 1448, 1125, 451 cm^{-1}. **MS** (ESI) *m/z* (relative intensity): 969

(50), 474 (100) [M+H]$^+$, 440 (10). **HR-MS** (ESI) *m/z* calcd for $C_{23}H_{23}F_3FeNOS$ [M+H]$^+$ 474.0798, found 474.0797.

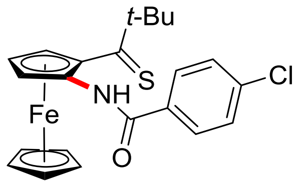

4-Chloro-*N*-[2-(2,2-dimethylpropanethioyl)ferrocenyl]benzamide (**3ae**)

The general procedure **A** was followed using 2,2-dimethyl-1-ferrocenylpropane-1-thione (**1a**) (71 mg, 0.25 mmol) and 3-(4-chlorophenyl)-1,4,2-dioxazol-5-one (**2e**) (59 mg, 0.3 mmol). Purification by column chromatography (*n*-hexane/EtOAc: 19/1) yielded **3ae** (85 mg, 77%) as a blue foam. **M. p.** = 89–90 °C. **^{1}H NMR** (300 MHz, $CDCl_3$): δ = 11.73 (s, 1H), 7.95 (d, J = 8.7 Hz, 2H), 7.50 (d, J = 8.7 Hz, 2H), 6.41 (dd, J = 3.0, 1.4 Hz, 1H), 4.85 (dd, J = 3.0, 1.4 Hz, 1H), 4.69 (t, J = 3.0 Hz, 1H), 4.11 (s, 5H), 1.56 (s, 9H). **^{13}C NMR** (75 MHz, $CDCl_3$): δ = 261.9 (C_q), 165.1 (C_q), 138.0 (C_q), 133.1 (C_q), 129.0 (CH), 128.8 (CH), 102.1 (C_q), 75.9 (C_q), 73.2 (CH), 68.7 (CH), 68.3 (CH), 66.5 (CH), 52.8 (C_q), 32.4 (CH_3). **IR** (ATR): 3040, 1559, 1437, 1343, 1165, 447 cm^{-1}. **MS** (ESI) *m/z* (relative intensity): 478 (100), 462 (40) [M+Na]$^+$, 440 (20) [M+H]$^+$. **HR-MS** (ESI) *m/z* calcd for $C_{22}H_{23}{}^{35}ClFeNOS$ [M+H]$^+$ 440.0532, found 440.0533.

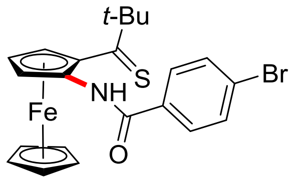

4-Bromo-*N*-[2-(2,2-dimethylpropanethioyl)ferrocenyl]benzamide (3af)

The general procedure **A** was followed using 2,2-dimethyl-1-ferrocenylpropane-1-thione (**1a**) (71 mg, 0.25 mmol) and 3-(4-bromophenyl)-1,4,2-dioxazol-5-one (**2f**) (73 mg, 0.3 mmol). Purification by column chromatography (*n*-hexane/EtOAc: 19/1) yielded **3af** (73 mg, 61%) as blue foam. **M. p.** = 96–97 °C. **^{1}H NMR** (300 MHz, $CDCl_3$): δ = 11.71 (s, 1H), 7.89 (d, J = 7.6 Hz, 2H), 7.67 (d, J = 7.6 Hz, 2H), 6.38 (dd, J = 3.0, 1.4 Hz, 1H), 4.83 (dd, J = 3.0, 1.4 Hz, 1H), 4.67 (t, J = 3.0 Hz, 1H), 4.09 (s, 5H), 1.54 (s, 9H). **^{13}C NMR** (75 MHz, $CDCl_3$) δ = 262.3 (C_q), 165.2 (C_q), 133.6 (C_q), 132.0 (C_q), 129.0 (CH), 126.5 (CH), 102.1 (C_q), 75.9 (C_q), 73.2 (CH), 68.7 (CH), 68.3 (CH), 66.5 (CH), 52.8 (C_q), 32.4 (CH_3). **IR** (ATR): 3077, 1579, 1507, 1000, 488 cm^{-1}. **MS (ESI)** *m/z* (relative intensity): 524 (100), 506 (30) [M+Na]$^+$, 484

(20) [M+H]$^+$, 402 (40). **HR-MS (ESI)** *m/z* calcd for $C_{22}H_{23}{}^{79}BrFeNOS$ [M+H]$^+$ 484.0029 found 484.0030; and $C_{22}H_{23}{}^{81}BrFeNOS$ [M+H]$^+$ 486.0008 found 486.0013.

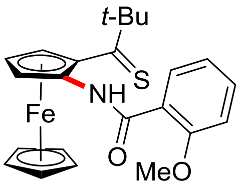

***N*-[2-(2,2-Dimethylpropanethioyl)ferrocenyl]-2-methoxybenzamide** (**3ag**)

The general procedure **A** was followed using 2,2-dimethyl-1-ferrocenylpropane-1-thione (**1a**) (71 mg, 0.25 mmol) and 3-(2-methoxyphenyl)-1,4,2-dioxazol-5-one (**2g**) (58 mg, 0.3 mmol). Purification by column chromatography (*n*-hexane/EtOAc: 10/1) yielded **3ag** (72 mg, 66%) as a blue foam. **M. p.** = 106–108 °C. ^{1}**H NMR** (400 MHz, $CDCl_3$): δ = 11.21 (s, 1H), 8.13 (dd, *J* = 7.8, 1.8 Hz, 1H), 7.48–7.43 (m, 1H), 7.06 (dd, *J* = 7.8 Hz, 7.8 Hz, 1H), 7.00 (d, *J* = 7.8 Hz, 1H), 6.17 (dd, *J* = 2.7, 1.4 Hz, 1H), 4.68 (dd, *J* = 2.7, 1.4 Hz, 1H), 4.53 (dd, *J* = 2.7, 2.7 Hz, 1H), 4.08 (s, 3H), 4.04 (s, 5H), 1.44 (s, 9H). ^{13}**C NMR** (100 MHz, $CDCl_3$): δ = 261.2 (C_q), 164.5 (C_q), 157.2 (C_q), 132.8 (CH), 132.0 (CH), 122.7 (C_q), 121.0 (CH), 111.2 (CH), 101.9 (C_q), 79.3 (C_q), 73.3 (CH), 69.2 (CH), 67.3 (CH), 64.2 (CH), 55.6 (CH_3), 52.5 (C_q), 31.8 (CH_3). **IR** (ATR): 3048, 1611, 1491, 805, 532 cm^{-1}. **MS** (ESI) *m/z* (relative intensity): 474 (100), 458 (40) [M+Na]$^+$, 436 (50) [M+H]$^+$, 174 (20). **HR-MS** (ESI) *m/z* calcd for $C_{23}H_{26}FeNO_2S$ [M+H]$^+$ 436.1029, found 436.1028.

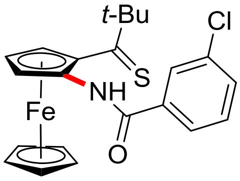

3-Chloro-*N*-[2-(2,2-dimethylpropanethioyl)ferrocenyl]benzamide (**3ah**)

The general procedure **A** was followed using 2,2-dimethyl-1-ferrocenylpropane-1-thione (**1a**) (71 mg, 0.25 mmol) and 3-(3-chlorophenyl)-1,4,2-dioxazol-5-one (**2h**) (59 mg, 0.3 mmol). Purification by column chromatography (*n*-hexane/EtOAc: 19/1) yielded **3ah** (80 mg, 73%) as a blue foam. **M. p.** = 98–100 °C. ^{1}**H NMR** (300 MHz, $CDCl_3$): δ = 11.72 (brs, 1H), 8.00 (t, *J* = 1.9 Hz, 1H), 7.85 (dt, *J* = 7.8, 1.4 Hz, 1H), 7.56–7.49 (m, 1H), 7.44 (t, *J* = 7.8 Hz, 1H), 6.38 (dd, *J* = 3.0, 1.4 Hz, 1H), 4.83 (dd, *J* = 3.0, 1.4 Hz, 1H), 4.67 (t, *J* = 3.0 Hz, 1H), 4.10 (s, 5H), 1.54 (s, 9H). ^{13}**C NMR** (100 MHz, $CDCl_3$): δ = 262.0 (C_q), 164.9 (C_q), 136.6 (C_q), 135.1 (C_q), 131.8 (CH), 130.1 (CH), 128.0 (CH), 125.4 (CH), 102.1 (C_q), 76.0 (C_q), 73.3 (CH), 68.9 (CH), 68.4 (CH), 66.7 (CH), 53.0 (C_q), 32.5 (CH_3). **IR** (ATR): 2956, 2921, 1670, 1526, 1400, 1251,

739 cm^{-1}. **MS** (ESI) *m/z* (relative intensity): 901 (30), 462 (50) [M+Na]$^+$, 390 (80). **HR-MS** (ESI) *m/z* calcd for $C_{22}H_{23}{}^{35}ClFeNOS$ [M+H]$^+$ 440.0533, found 440.0538.

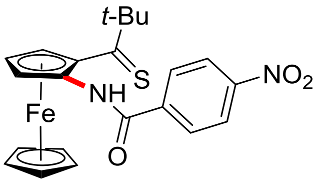

***N*-[2-(2,2-Dimethylpropanethioyl)ferrocenyl]-4-nitrobenzamide** (**3ai**)

The general procedure **A** was followed using 2,2-dimethyl-1-ferrocenylpropane-1-thione (**1a**) (71 mg, 0.25 mmol) and 3-(4-nitrophenyl)-1,4,2-dioxazol-5-one (**2i**) (62 mg, 0.3 mmol). Purification by column chromatography (*n*-hexane/EtOAc: 19/1) yielded **3ai** (90 mg, 80%) as a blue foam. **M. p.** = 90–92 °C. ^{1}**H NMR** (400 MHz, $CDCl_3$): δ = 11.96 (brs, 1H), 8.37 (d, *J* = 8.8 Hz, 2H), 8.15 (d, *J* = 8.8 Hz, 2H), 6.39 (dd, *J* = 3.0, 1.4 Hz, 1H), 4.87 (dd, *J* = 3.0, 1.4 Hz, 1H), 4.71 (t, *J* = 3.0 Hz, 1H), 4.12 (s, 5H), 1.55 (s, 9H). ^{13}**C NMR** (100 MHz, $CDCl_3$): δ = 262.3 (C_q), 164.1 (C_q), 149.8 (C_q), 140.3 (C_q), 128.6 (CH), 124.1 (CH), 101.6 (C_q), 76.0 (C_q), 73.4 (CH), 69.1 (CH), 68.5 (CH), 67.0 (CH), 53.1 (C_q), 32.5 (CH_3). **IR** (ATR): 2957, 2921, 1672, 1527, 1313, 1065, 753 cm^{-1}. **MS** (ESI) *m/z* (relative intensity): 923 (30), 473 (50) [M+Na]$^+$, 451 (100) [M+H]$^+$, 381 (80). **HR-MS** (ESI) *m/z* calcd for $C_{22}H_{23}FeN_2O_3S$ [M+H]$^+$ 451.0774, found 451.0774.

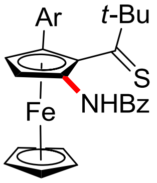

Ar = 4-MeC_6H_4

***N*-{2-(2,2-Dimethylpropanethioyl)-4'-methyl-[1,1'-ferrocenylphenyl]-3-yl}benzamide (3ba)**

The general procedure **A** was followed using 2,2-dimethyl-1-(4'-methyl-[1,1'-ferrocenephenyl]-2-yl)propane-1-thione (**1b**) (94 mg, 0.25 mmol) and 3-phenyl-1,4,2-dioxazol-5-one (**2a**) (49 mg, 0.3 mmol). Purification by column chromatography (*n*-hexane/EtOAc: 19/1) yielded **3ba** (66 mg, 53%) as a red foam. **M. p.** = 108–110 °C. ^{1}**H NMR** (400 MHz, $CDCl_3$): 7.73–7.65 (m, 2H), 7.58 (brs, 1H), 7.55–7.43 (m, 3H), 7.37 (d, *J* = 8.1 Hz, 2H), 7.13–7.03 (m, 2H), 5.45 (d, *J* = 2.6 Hz, 1H), 4.64 (d, *J* = 2.6 Hz, 1H), 4.15 (s, 5H), 2.33 (s, 3H), 1.03 (s, 9H). ^{13}**C NMR** (100 MHz, $CDCl_3$): δ = 272.2 (C_q), 165.6 (C_q), 136.6 (C_q), 136.2 (C_q), 134.3 (C_q), 132.0 (CH), 129.1 (CH), 129.0 (CH), 128.3 (CH), 126.9

(CH), 98.0 (C_q), 96.9 (C_q), 80.6 (C_q), 73.7 (CH), 65.0 (CH), 62.3 (CH), 56.0 (C_q), 32.1 (CH_3), 21.3 (CH_3). **IR** (ATR): 2956, 2921, 1675, 1520, 1482, 818 cm^{-1}. **MS** (ESI) *m/z* (relative intensity): 518 (100) $[M+Na]^+$, 496 (40) $[M+H]^+$, 398 (20). **HR-MS** (ESI) *m/z* calcd for $C_{29}H_{30}FeNOS$ $[M+H]^+$ 496.1392, found 496.1387.

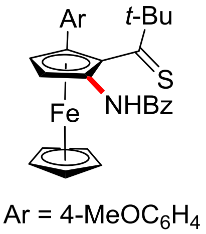

***N*-{2-(2,2-Dimethylpropanethioyl)-4'-(methoxy)-[1,1'-ferrocenephenyl]-3-yl}benzamide** (**3ca**)

The general procedure **A** was followed using 2,2-dimethyl-1-{4'-(methoxy)-[1,1'-ferrocenephenyl]-2-yl}propane-1-thione (**1c**) (98 mg, 0.25 mmol) and 3-phenyl-1,4,2-dioxazol-5-one (**2a**) (49 mg, 0.3 mmol). Purification by column chromatography (*n*-hexane/EtOAc: 19/1) yielded **3ca** (77 mg, 60%) as a red foam. **M. p.** = 105–107 °C. ^{1}**H NMR** (400 MHz, $CDCl_3$): δ = 7.80–7.65 (m, 2H), 7.61–7.35 (m, 6H), 6.82 (d, J = 8.7 Hz, 2H), 5.43 (d, J = 2.6 Hz, 1H), 4.61 (d, J = 2.6 Hz, 1H), 4.16 (s, 5H), 3.82 (s, 3H), 1.02 (s, 9H). ^{13}**C NMR** (100 MHz, $CDCl_3$): δ = 272.2 (C_q), 165.7 (C_q), 158.7 (C_q), 134.3 (C_q), 132.0 (C_q), 131.3 (CH), 129.5 (CH), 129.0 (CH), 126.9 (CH), 113.9 (CH), 97.8 (C_q), 96.7 (C_q), 73.6 (CH), 72.2 (C_q), 64.9 (CH), 62.4 (CH), 56.0 (C_q), 55.4 (CH_3), 32.0 (CH_3). **IR** (ATR): 2956, 2921, 1675, 1520, 1463, 1247, 1177, 755 cm^{-1}. **MS** (ESI) *m/z* (relative intensity): 534 (100) $[M+Na]^+$, 512 (80) $[M+H]^+$. **HR-MS** (ESI) *m/z* calcd for $C_{29}H_{30}FeNO_2S$ $[M+H]^+$ 512.1342, found 512.1347.

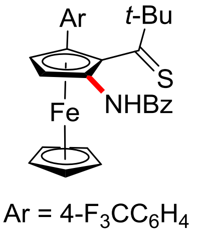

***N*-{2-(2,2-Dimethylpropanethioyl)-4'-(trifluoromethyl)-[1,1'-ferrocenephenyl]-3-yl}benzamide** (**3da**)

The general procedure **A** was followed using 2,2-dimethyl-1-{4'-(trifluoromethyl)-[1,1'-ferrocenephenyl]-2-yl}propane-1-thione (**1d**) (108 mg, 0.25 mmol) and 3-phenyl-1,4,2-dioxazol-5-one (**2a**) (49 mg, 0.3 mmol). Purification by column chromatography (*n*-

hexane/EtOAc: 19/1) yielded **3da** (70 mg, 51%) as a blue foam. **M. p.** = 102–104 °C. **^{1}H NMR** (400 MHz, $CDCl_3$): δ = 7.73–7.67 (m, 2H), 7.62–7.43 (m, 8H), 5.55 (d, J = 2.7 Hz, 1H), 4.68 (d, J = 2.7 Hz, 1H), 4.16 (s, 5H), 1.04 (s, 9H). **^{13}C NMR** (100 MHz, $CDCl_3$): δ = 271.2 (C_q), 165.6 (C_q), 144.0 (C_q), 134.1 (C_q), 132.2 (CH), 129.2 (q, $^{2}J_{C-F}$ = 32.6 Hz, C_q), 129.1 (CH), 128.6 (CH), 128.9 (CH), 126.9 (CH), 125.7 (q, $^{1}J_{C-F}$ = 273.6 Hz, C_q), 125.4 (q, $^{3}J_{C-F}$ = 3.8 Hz, CH), 98.5 (C_q), 97.3 (C_q), 73.9 (C_q), 65.6 (CH), 62.2 (CH), 56.3 (C_q), 32.0 (CH_3). **^{19}F NMR** (282 MHz, $CDCL_3$) δ = −62.47. **IR** (ATR): 2951, 2925, 1670, 1530, 1400, 1050, 732 cm^{-1}. **MS** (ESI) *m/z* (relative intensity): 572 (100) [M+Na]$^+$, 550 (40) [M+H]$^+$, 452 (30), 381 (20). **HR-MS** (ESI) *m/z* calcd for $C_{29}H_{27}FeNOSF_3$, [M+H]$^+$ 550.1110, found 550.1116.

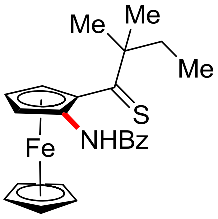

***N*-[2-(2,2-Dimethylbutanethioyl)ferrocenyl]benzamide** (**3ea**)

The general procedure **A** was followed using 2,2-dimethyl-1-ferrocenylbutane-1-thione (**1e**) (75 mg, 0.25 mmol) and 3-phenyl-1,4,2-dioxazol-5-one (**2a**) (49 mg, 0.3 mmol). Purification by column chromatography (*n*-hexane/EtOAc: 19/1) yielded **3ea** (85 mg, 81%) as blue foam. **M. p.** = 95–97 °C. **^{1}H NMR** (400 MHz, $CDCl_3$): δ = 11.51 (brs, 1H), 8.16–7.91 (m, 2H), 7.67–7.27 (m, 3H), 6.38 (dd, J = 2.9, 1.5 Hz, 1H), 4.80 (dd, J = 2.9, 1.5 Hz, 1H), 4.62 (t, J = 2.9 Hz, 1H), 4.09 (s, 5H), 2.10 (dd, J = 13.9, 7.4 Hz, 1H), 1.84 (dd, J = 13.9, 7.4 Hz, 1H), 1.56 (s, 3H), 1.41 (s, 3H), 0.78 (t, J = 7.5 Hz, 3H). **^{13}C NMR** (100 MHz, $CDCl_3$): δ = 262.2 (C_q), 166.2 (C_q), 134.8 (C_q), 131.8 (CH), 128.8 (CH), 127.5 (CH), 102.5 (C_q), 77.4 (C_q), 73.3 (CH), 68.4 (CH), 68.3 (CH), 65.2 (CH), 56.7 (C_q), 36.1 (CH_2), 30.3 (CH_3), 30.3 (CH_3), 9.3 (CH_3). **IR** (ATR): 2964, 2924, 1669, 1523, 1488, 1399, 1249, 650 cm^{-1}. **MS** (ESI) *m/z* (relative intensity): 442 (100) [M+Na]$^+$, 420 (80) [M+H]$^+$. **HR-MS** (ESI) *m/z* calcd for $C_{23}H_{26}FeNOS$ [M+H]$^+$ 420.1079, found 420.1077.

H/D Exchange experiment

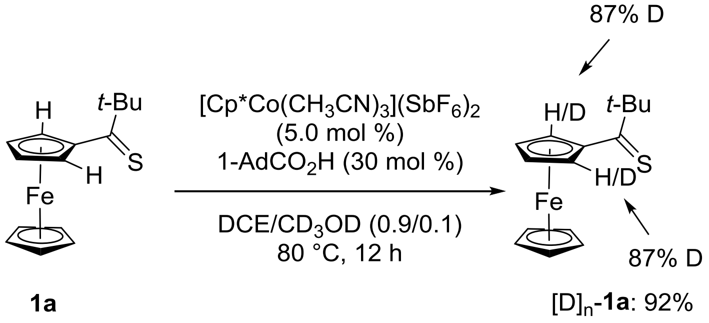

Thiocarbonylferrocene **1a** (71 mg, 0.25 mmol), 1-AdCO_2H (13.5 mg 30 mol %), [Cp*Co(CH_3CN)$_3$](SbF_6)$_2$ (6.9 mg, 5.0 mol %), DCE (0.9 mL) and CD_3OD (0.1 mL) were placed in a 25 mL Schlenk tube under N_2 and the mixture was stirred at 80 ºC for 12 h. At ambient temperature, the mixture was transferred into a round bottom flask with CH_2Cl_2 (5 mL) and concentrated in vacuo. Purification by column chromatography on silica gel (*n*-hexane) yielded the product [D]$_n$-**1a** (65 mg, 92%) with 85% deuterium incorporation.

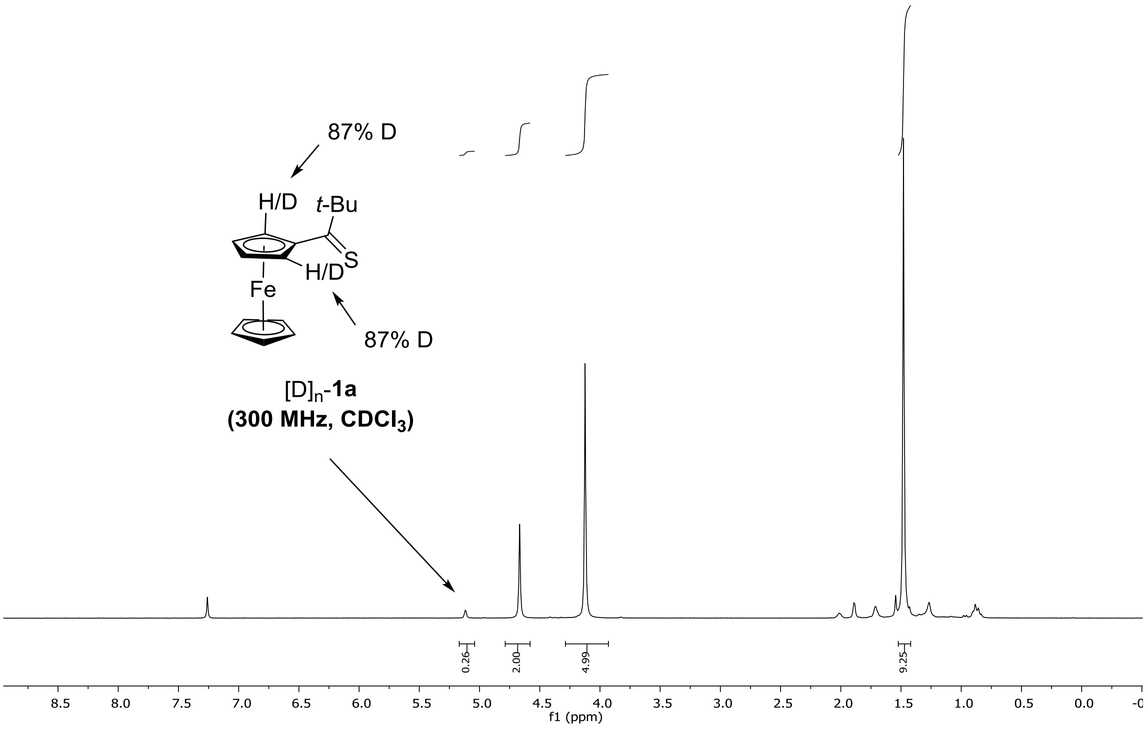

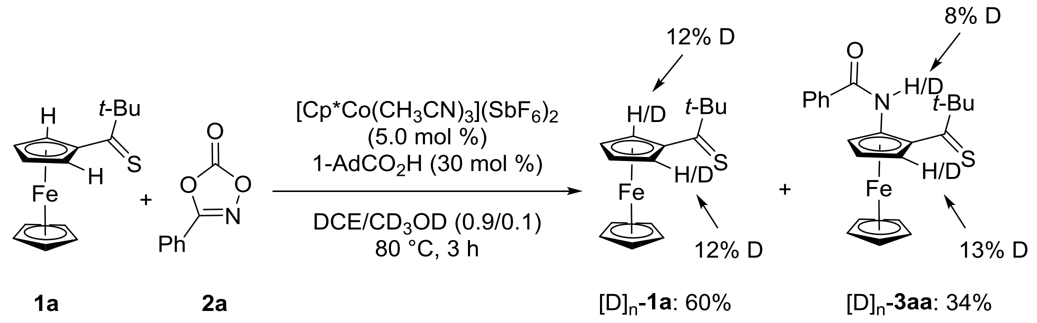

Thiocarbonylferrocene **1a** (71 mg, 0.25 mmol), dioxazolone **2a** (49 mg, 0.3 mmol, 1.2 equiv), 1-AdCO_2H (13.5 mg 30 mol %), [Cp*Co(CH_3CN)$_3$](SbF_6)$_2$ (6.9 mg, 5.0 mol %), DCE (0.9 mL) and CD_3OD (0.1 mL) were placed in a 25 mL Schlenk tube under N_2 and the mixture was stirred at 80 ºC for 3 h. At ambient temperature, the mixture was transferred into a round bottom flask with CH_2Cl_2 (15 mL) and concentrated in vacuo. Purification by column chromatography on silica gel (*n*-hexane/EtOAc: 19/1) yielded the product [D]$_n$-**1a** (65 mg, 60%) with 12% deuterium incorporation and [D]$_n$-**3aa** (34 mg, 34%) with 13% deuterium incorporation in *ortho*-ferrocene and 8% deuterium incorporation in amide, respectively.

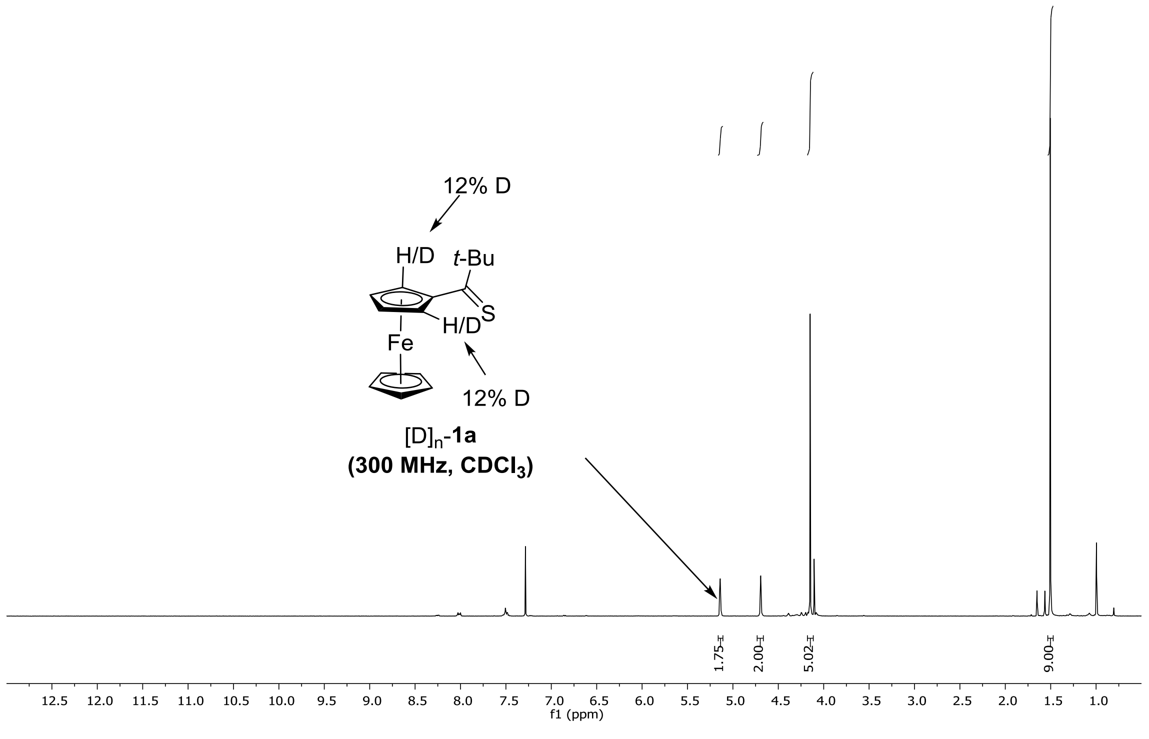

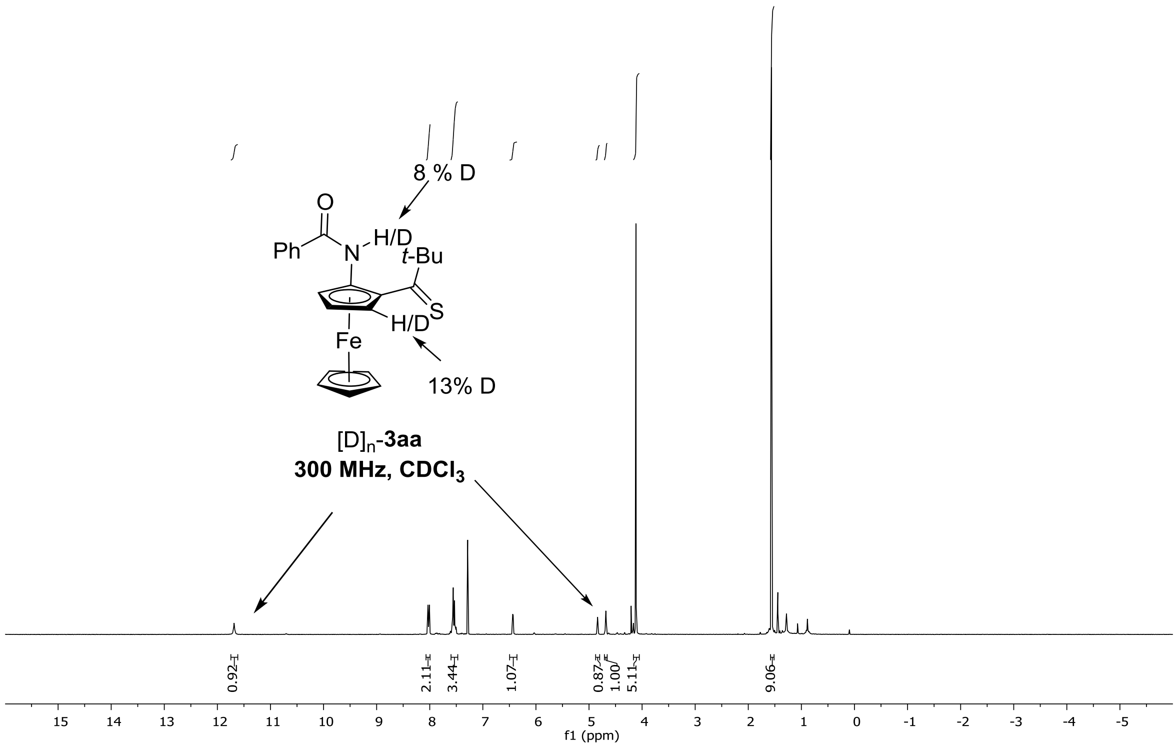

Intermolecular competition experiment

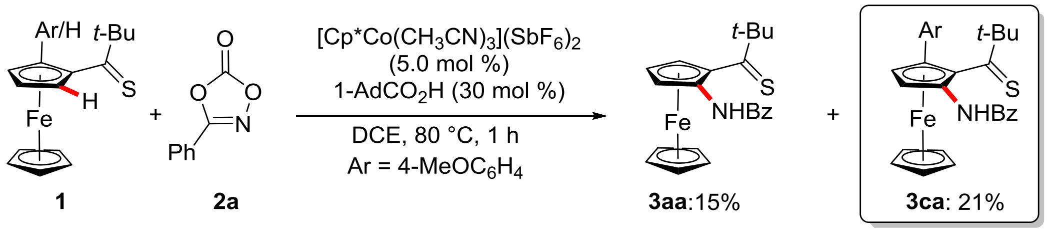

A suspension of 2,2-dimethyl-1-ferrocenylpropane-1-thione (**1a**) (71 mg, 0.25 mmol), 2,2-dimethyl-1-{4'-(methoxy)-[1,1'-ferrocenephenyl]-2-yl}propane-1-thione (**1c**) (91 mg, 0.25 mmol), 3-phenyl-1,4,2-dioxazol-5-one (**2a**) (49 mg, 0.3 mmol), 1-$AdCO_2H$ (13.5 mg, 30 mol %) and $[Cp^*Co(CH_3CN)_3](SbF_6)_2$ (6.9 mg, 5.0 mol %) in DCE (1.0 mL) was stirred at 80 ºC for 1 h under N_2 atmosphere. At ambient temperature, the mixture was transferred into a round-bottom flask with CH_2Cl_2 (15 mL) and concentrated in vacuo. Purification by column chromatography (*n*-hexane/EtOAc: 19/1) on silica gel afforded the products **3aa** (15 mg, 15%) and **3ca** (27 mg, 21%).

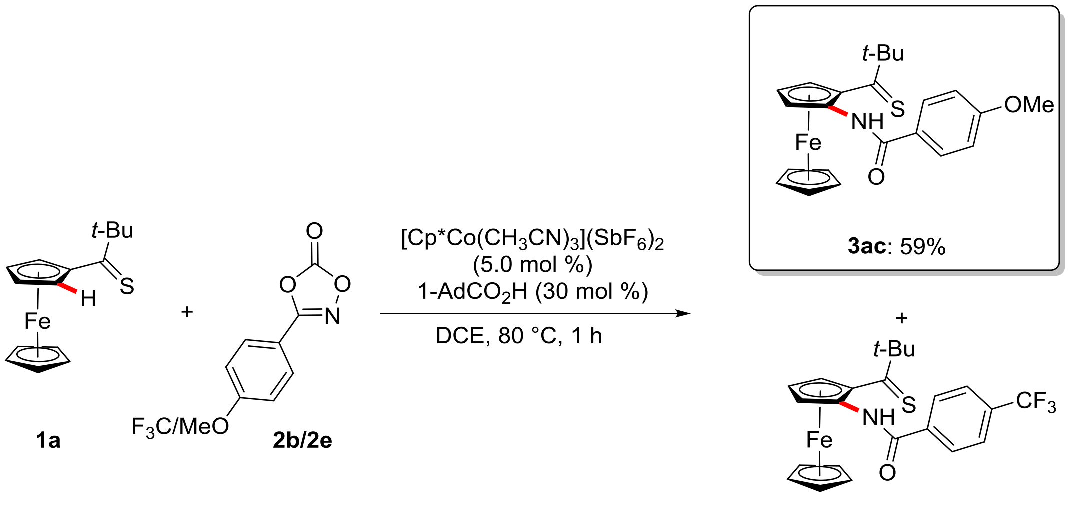

A suspension of 2,2-dimethyl-1-ferrocenylpropane-1-thione (**1a**) (71 mg, 0.25 mmol), 3-(4-methoxyphenyl)-1,4,2-dioxazol-5-one (**2b**) (48 mg, 0.25 mmol), 3-[4-(trifluoromethyl) phenyl]-1,4,2-dioxazol-5-one (**2e**) (58 mg, 0.25 mmol), 1-AdCO$_2$H (13.5 mg, 30 mol %) and [Cp*Co(CH$_3$CN)$_3$](SbF$_6$)$_2$ (6.9 mg, 5.0 mol %) in DCE (1.0 mL) was stirred at 80 ºC for 1 h under N$_2$ atmosphere. After cooling to ambient temperature, the mixture was transferred into a round-bottom flask with CH$_2$Cl$_2$ (15 mL) and concentrated in vacuo. Purification by column chromatography (*n*-hexane/EtOAc: 19/1) on silica gel afforded the products **3ab** (64 mg, 59%) and **3ae** (35 mg, 30%).

Late-stage diversification

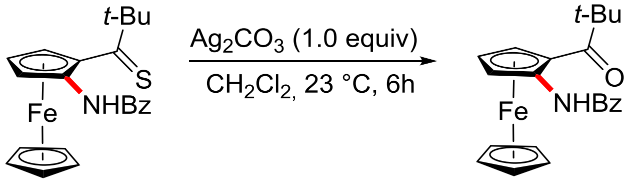

A suspension of *N*-[2-(2,2-dimethylpropanethioyl)ferrocenyl]benzamide (**3aa**) (101 mg, 0.25 mmol) and Ag_2CO_3 (69 mg, 0.25 mmol) in CH_2Cl_2 (1.0 mL) was stirred at 23 ºC for 6 h under air. Then, the mixture was transferred into a round bottom flask and concentrated in vacuo. Purification by column chromatography on silica gel (*n*-hexane/EtOAc: 19/1) afforded the product **4aa** as a yellow oil (97%). **^{1}H NMR** (300 MHz, $CDCl_3$): δ = 10.68 (s, 1H), 8.19–7.92 (m, 2H), 7.52–7.32 (m, 3H), 6.01 (s, 1H), 4.65 (dd, *J* = 2.8, 1.5 Hz, 1H), 4.44 (t, *J* = 2.8 Hz, 1H), 4.18 (s, 5H), 1.42 (s, 9H). **^{13}C NMR** (100 MHz, $CDCl_3$): δ = 217.4 (C_q), 165.7 (C_q), 134.5 (C_q), 131.8 (CH), 128.8 (CH), 127.2 (CH), 100.4 (C_q), 71.1 (CH), 68.4 (CH), 66.6 (CH), 65.0 (CH), 63.7 (C_q), 45.5 (C_q), 28.6 (CH_3). **IR** (ATR): 2957, 2921, 1671, 1530, 701 cm^{-1}. **MS** (ESI) *m/z* (relative intensity): 412 (100) $[M+Na]^+$, 406 (40), 390 (30), 381 (20). **HR-MS** (ESI) *m/z* calcd for $C_{22}H_{24}FeNO_2$ $[M+H]^+$ 390.1151, found 390.1151.

References

[1] Cai, Z.-J.; Liu, C.-X.; Gu, Q.; You, S.-L. *Angew. Chem. Int. Ed.* **2018**, *57*, 1296-1299.

[2] Bizet, V.; Buglioni, L.; Bolm, C. *Angew. Chem. Int. Ed*. **2014**, *53*, 5639-5642.

[3] Yu, D. G.; Gensch, T. F.; S. de Azambuja, Vásquez-Céspedes.; Glorius, F. *J. Am. Chem. Soc*. **2014**, *136*, 17722-17725.

^{1}H, ^{13}C and ^{19}F NMR spectra

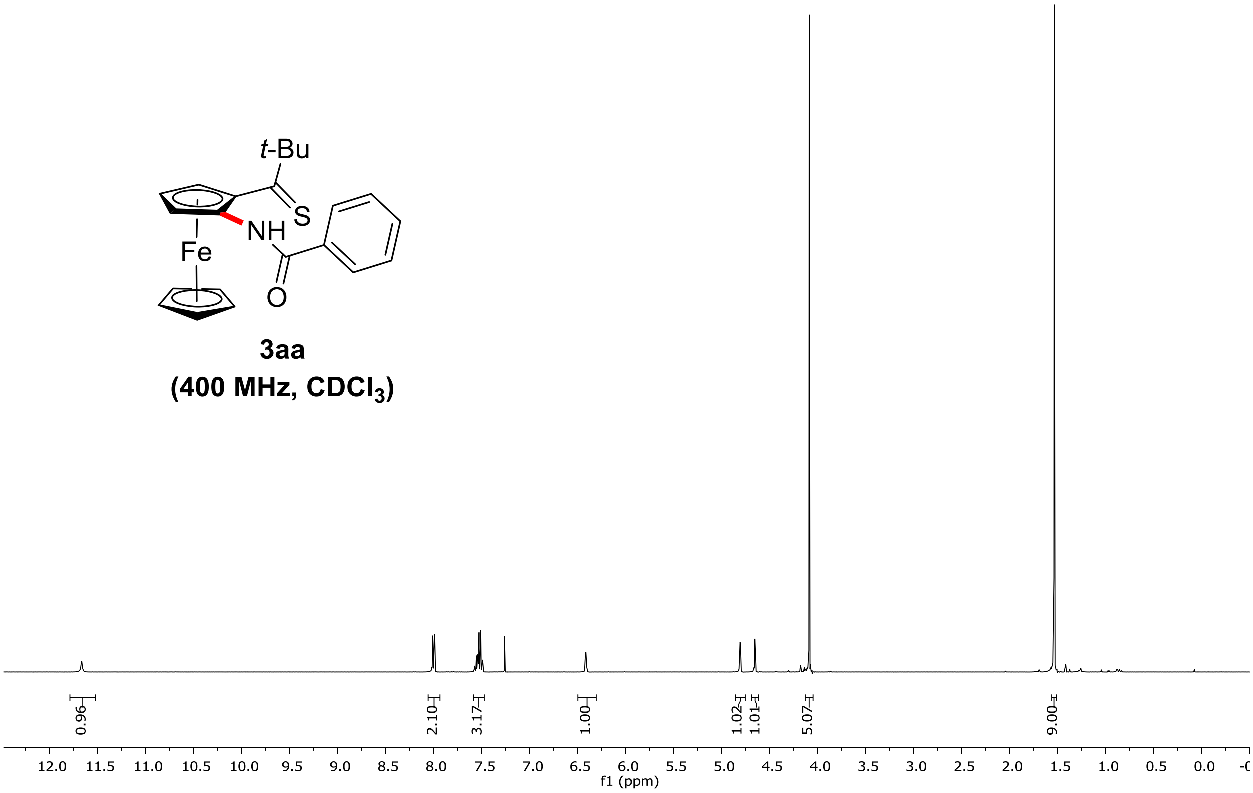

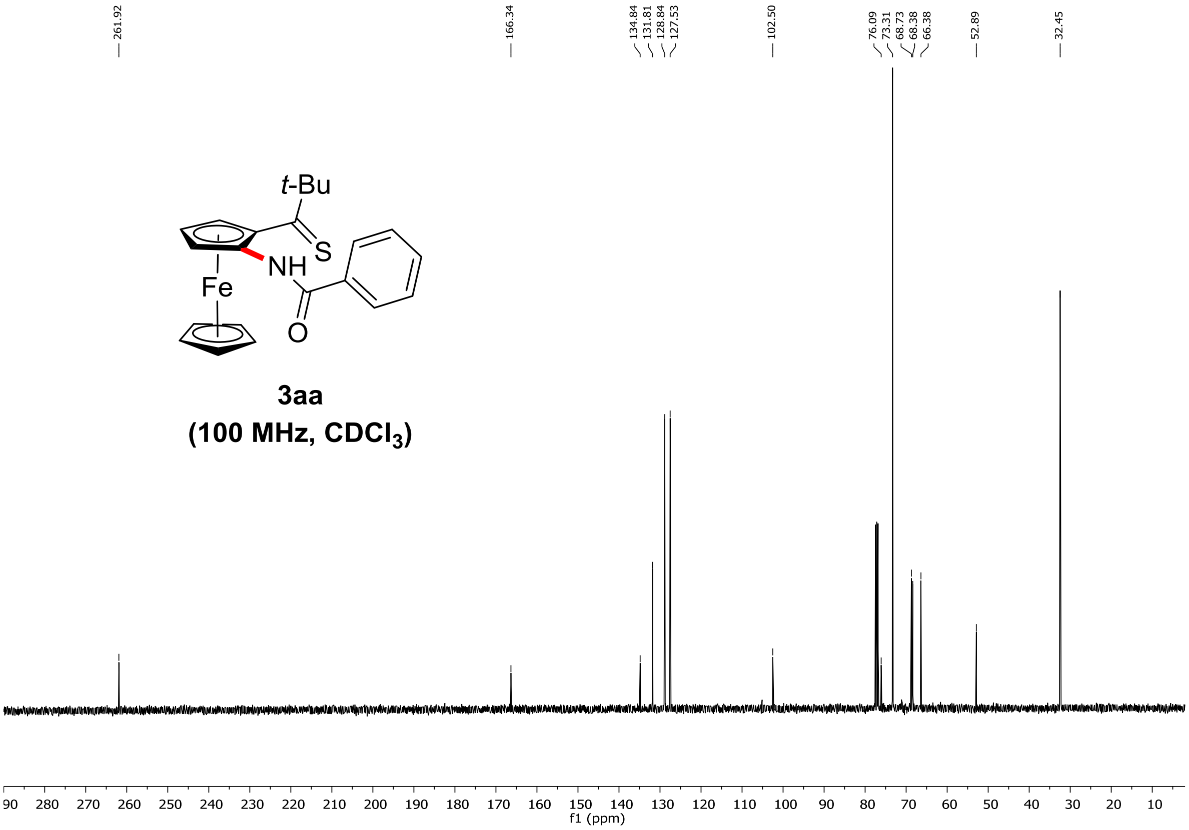

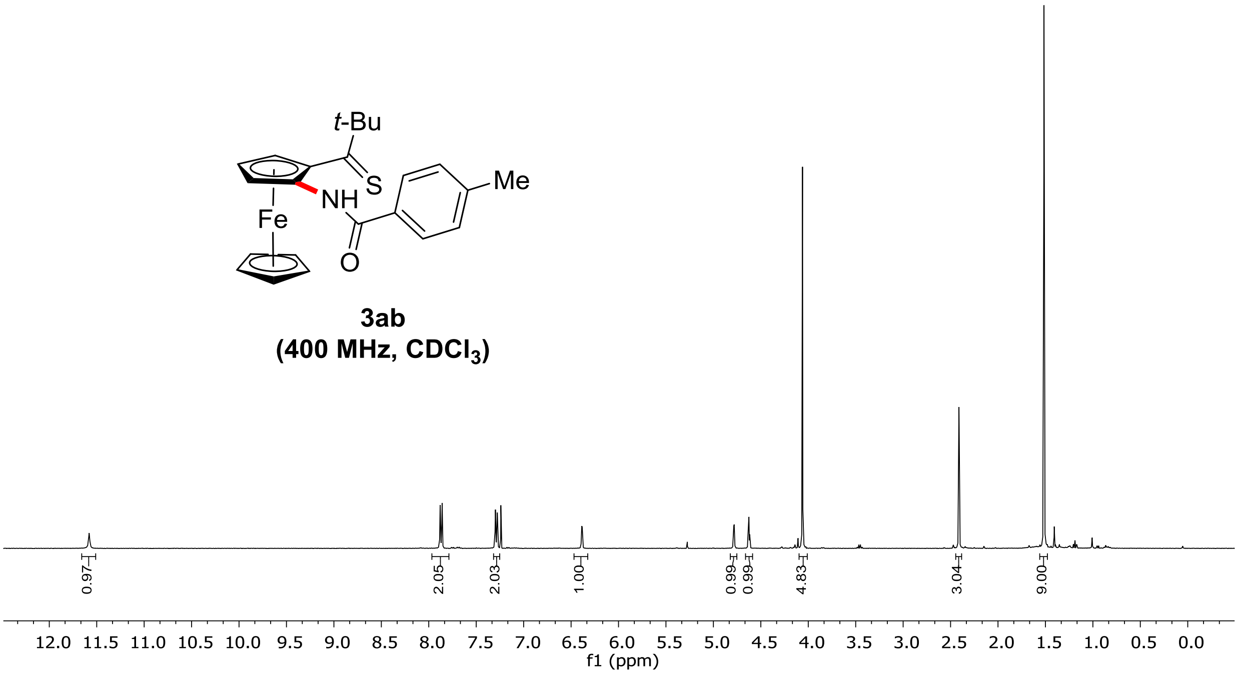
t-Bu
S
NH
Me
Fe
O
3ab
(400 MHz, CDCl3)
0.97
2.05
2.03
1.00
0.99
0.99
4.83
3.04
9.00
12.0 11.5 11.0 10.5 10.0 9.5 9.0 8.5 8.0 7.5 7.0 6.5 6.0 5.5 5.0 4.5 4.0 3.5 3.0 2.5 2.0 1.5 1.0 0.5 0.0
f1 (ppm)

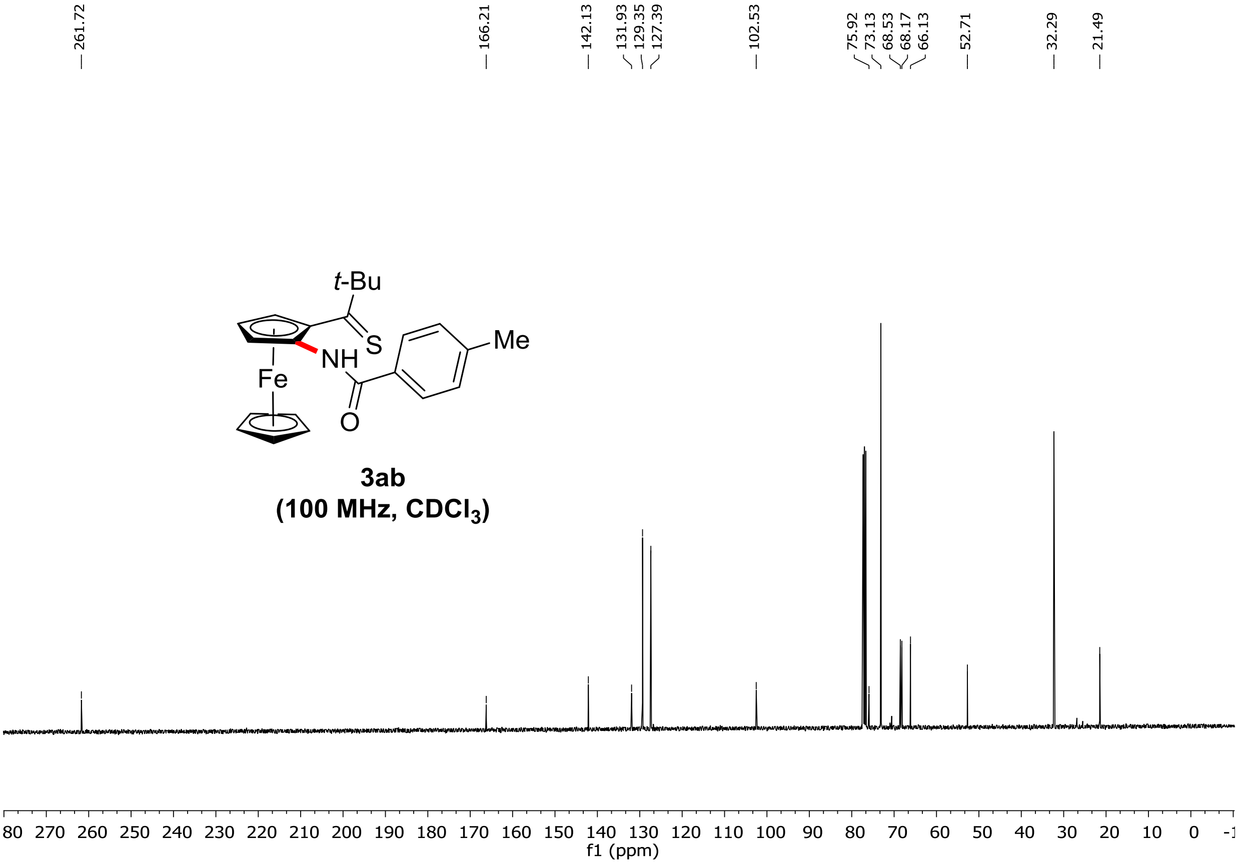
261.72
166.21
142.13
131.93
129.35
127.39
102.53
75.92
73.13
68.53
68.17
66.13
52.71
32.29
21.49
t-Bu
S
NH
Me
Fe
O
3ab
(100 MHz, CDCl3)
80 270 260 250 240 230 220 210 200 190 180 170 160 150 140 130 120 110 100 90 80 70 60 50 40 30 20 10 0
f1 (ppm)

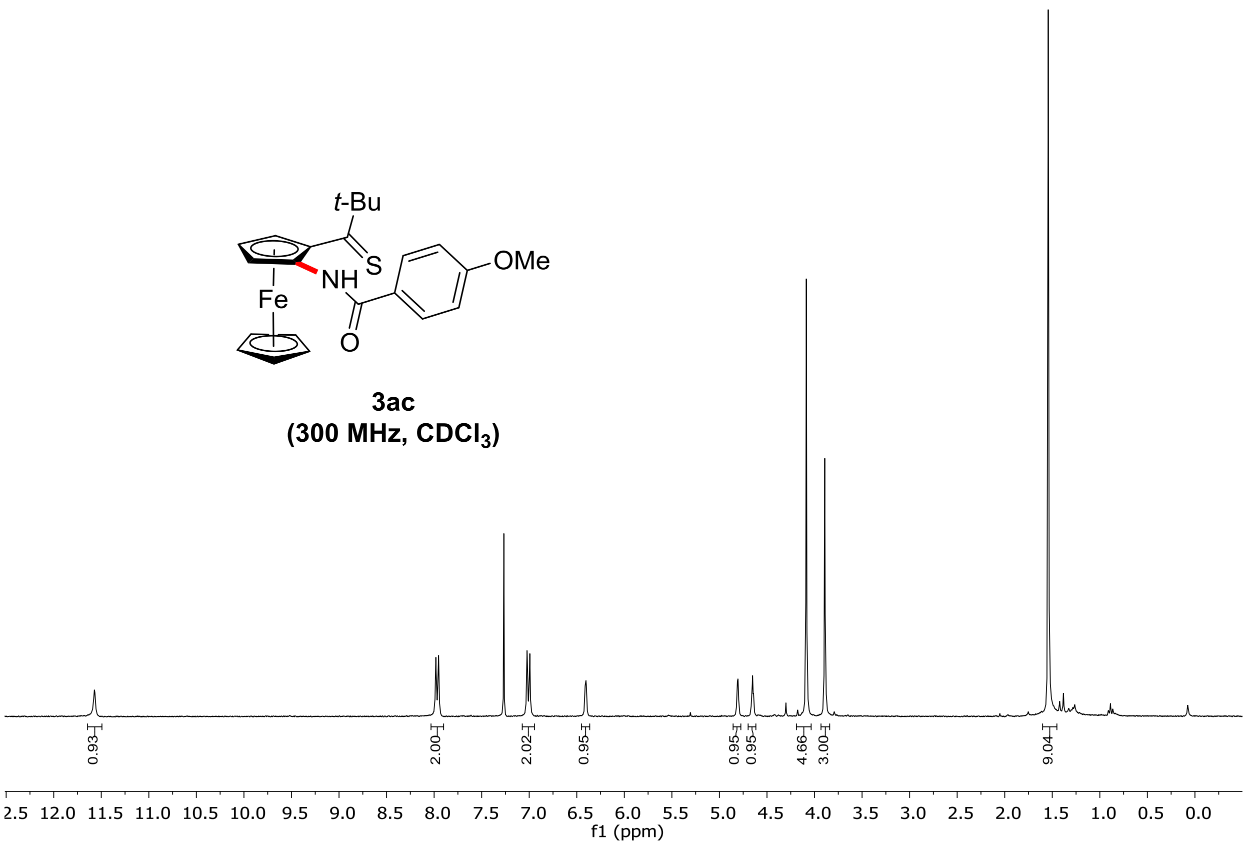
t-Bu
S
NH
Fe
O
OMe
3ac
(300 MHz, CDCl3)
0.93
2.00
2.02
0.95
0.95
0.95
4.66
3.00
9.04
2.5 12.0 11.5 11.0 10.5 10.0 9.5 9.0 8.5 8.0 7.5 7.0 6.5 6.0 5.5 5.0 4.5 4.0 3.5 3.0 2.5 2.0 1.5 1.0 0.5 0.0
f1 (ppm)

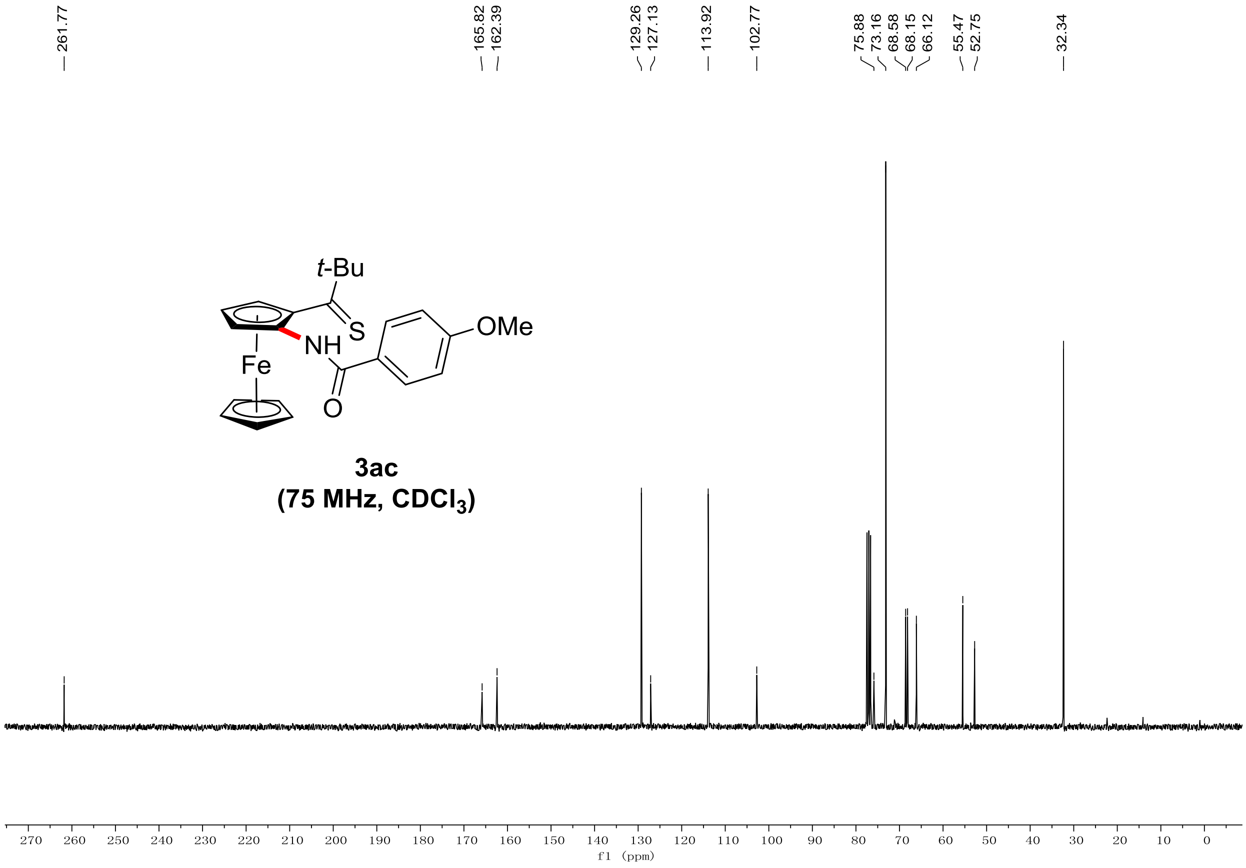
261.77
165.82
162.39
129.26
127.13
113.92
102.77
75.88
73.16
68.58
68.15
66.12
55.47
52.75
32.34
t-Bu
S
NH
Fe
O
OMe
3ac
(75 MHz, CDCl3)
270 260 250 240 230 220 210 200 190 180 170 160 150 140 130 120 110 100 90 80 70 60 50 40 30 20 10 0
f1 (ppm)

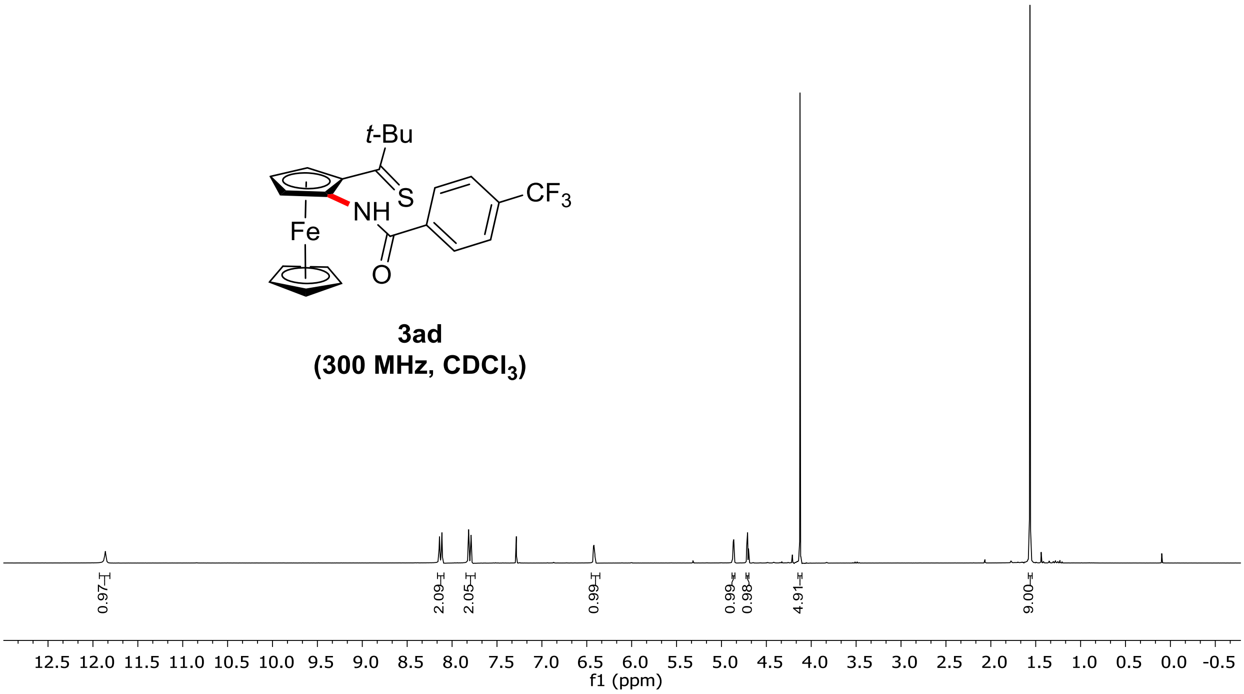
t-Bu
S
NH
CF3
Fe
O
3ad
(300 MHz, CDCl3)
0.97
2.09
2.05
0.99
0.99
0.98
4.91
9.00
f1 (ppm)

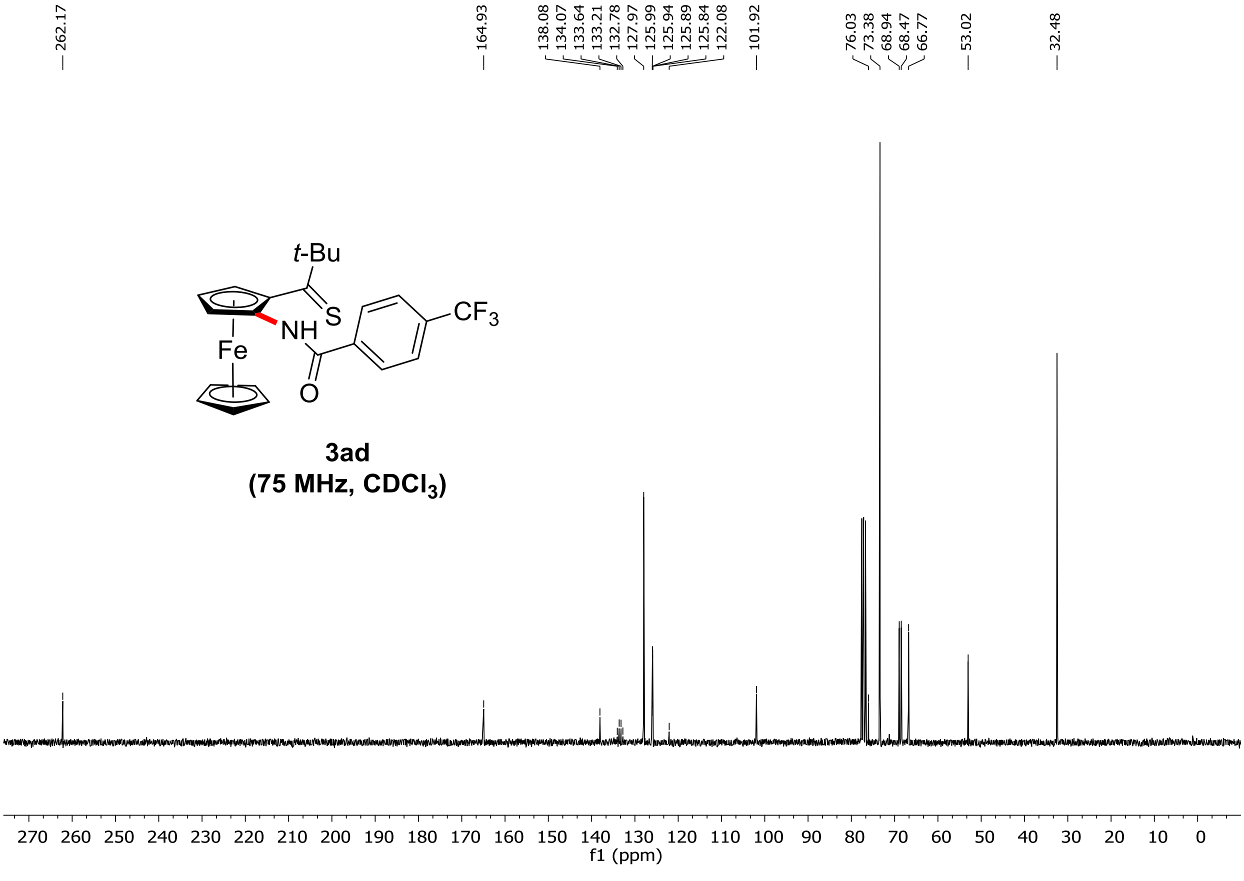
262.17
164.93
138.08
134.07
133.64
133.21
132.78
127.97
125.99
125.94
125.89
125.84
122.08
101.92
76.03
73.38
68.94
68.47
66.77
53.02
32.48
t-Bu
S
NH
CF3
Fe
O
3ad
(75 MHz, CDCl3)
f1 (ppm)

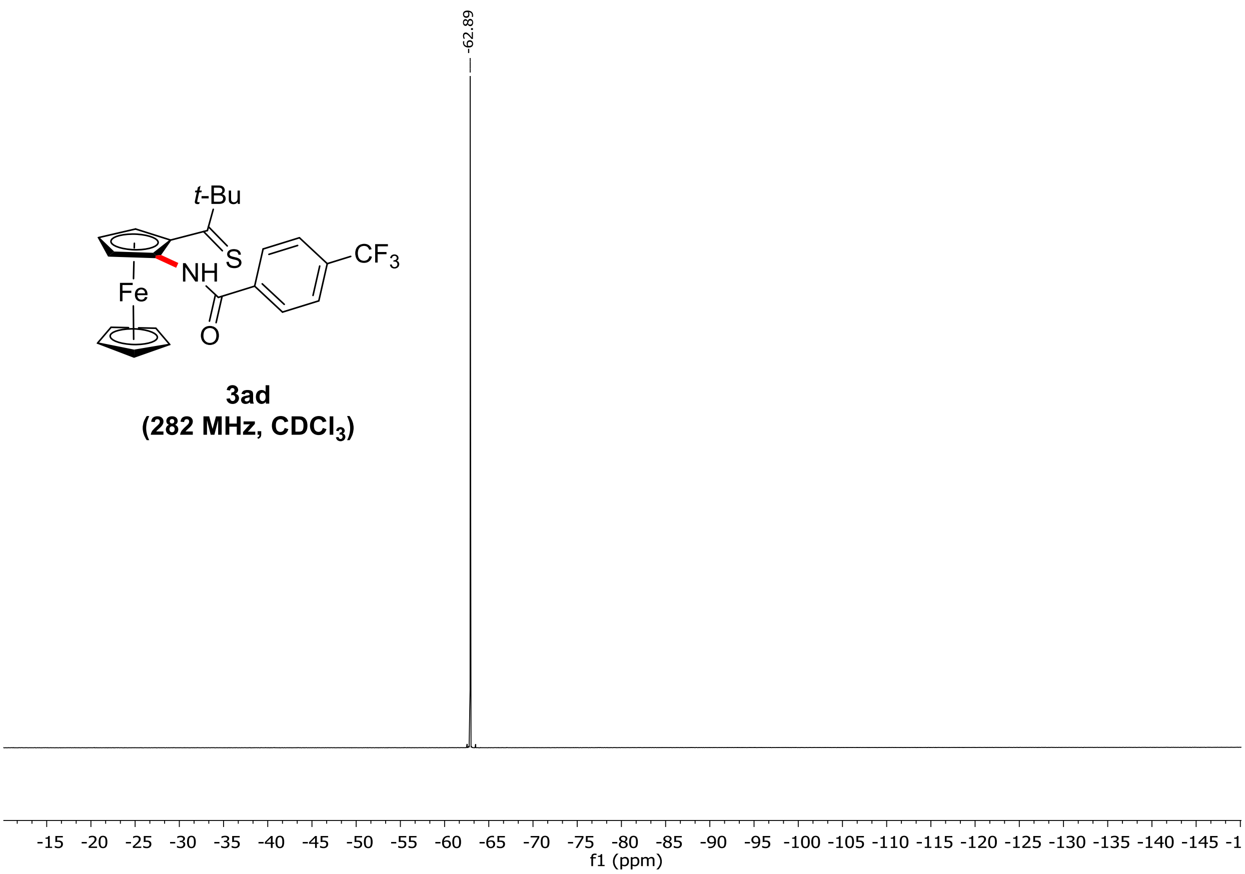
-62.89
t-Bu
S
NH
CF3
Fe
O
3ad
(282 MHz, CDCl3)
-15 -20 -25 -30 -35 -40 -45 -50 -55 -60 -65 -70 -75 -80 -85 -90 -95 -100 -105 -110 -115 -120 -125 -130 -135 -140 -145 -1
f1 (ppm)

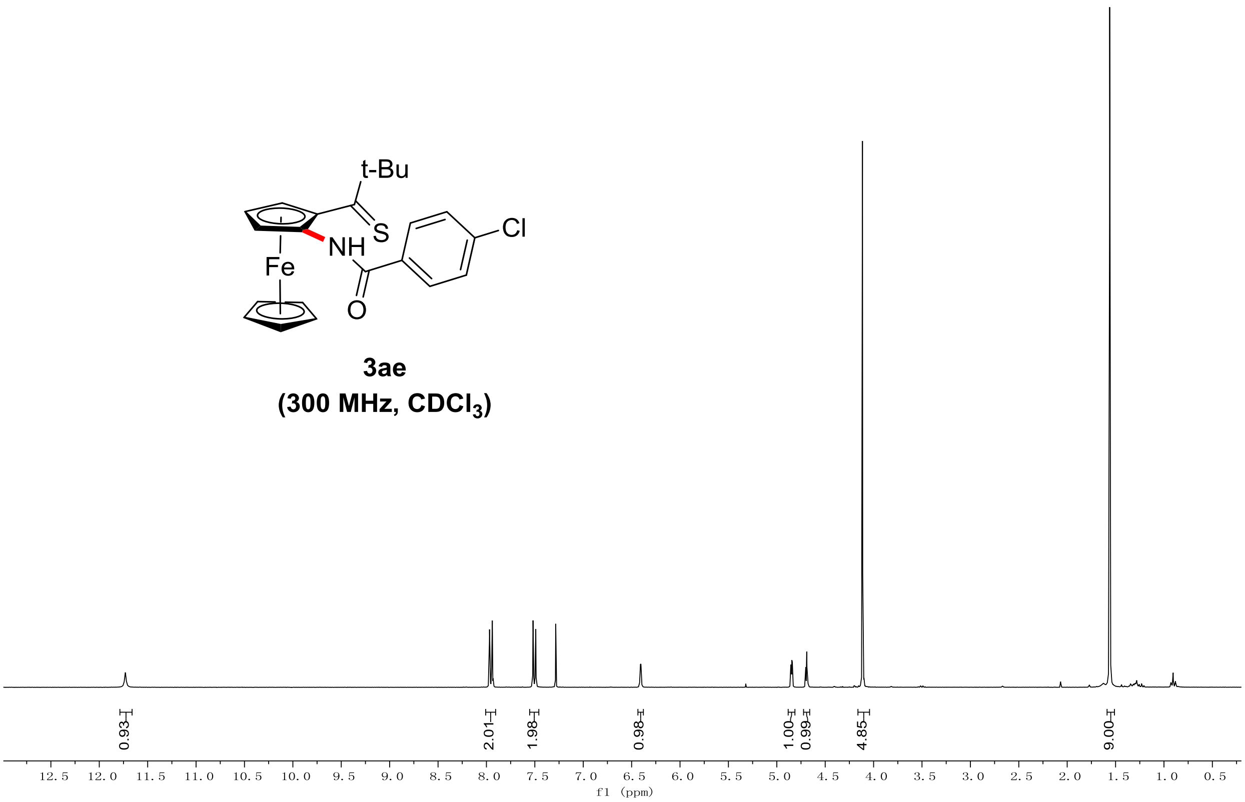
t-Bu
S
NH
Cl
Fe
O
3ae
(300 MHz, CDCl3)
0.93
2.01
1.98
0.98
1.00
0.99
4.85
9.00
12.5 12.0 11.5 11.0 10.5 10.0 9.5 9.0 8.5 8.0 7.5 7.0 6.5 6.0 5.5 5.0 4.5 4.0 3.5 3.0 2.5 2.0 1.5 1.0 0.5
f1 (ppm)

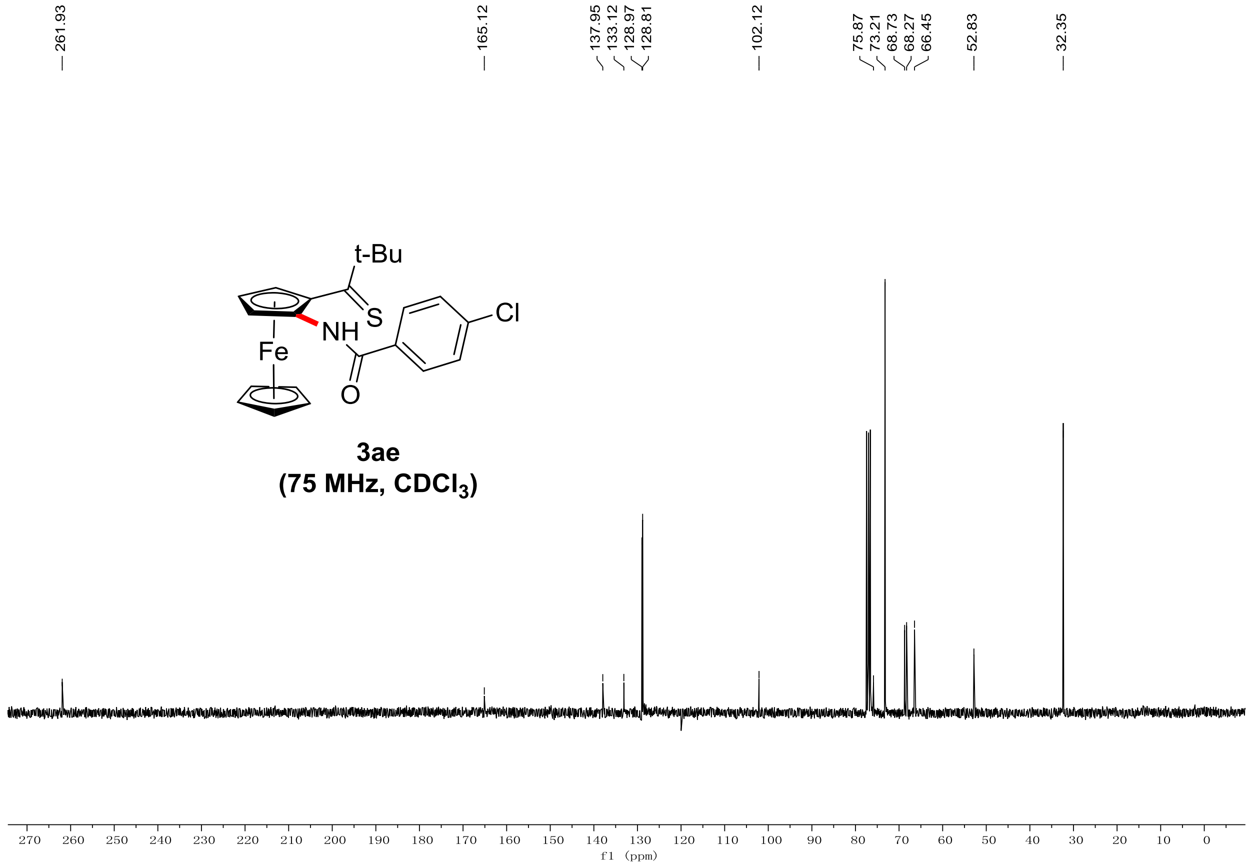
261.93
165.12
137.95
133.12
128.97
128.81
102.12
75.87
73.21
68.73
68.27
66.45
52.83
32.35
t-Bu
S
NH
Cl
Fe
O
3ae
(75 MHz, CDCl3)
270 260 250 240 230 220 210 200 190 180 170 160 150 140 130 120 110 100 90 80 70 60 50 40 30 20 10 0
f1 (ppm)

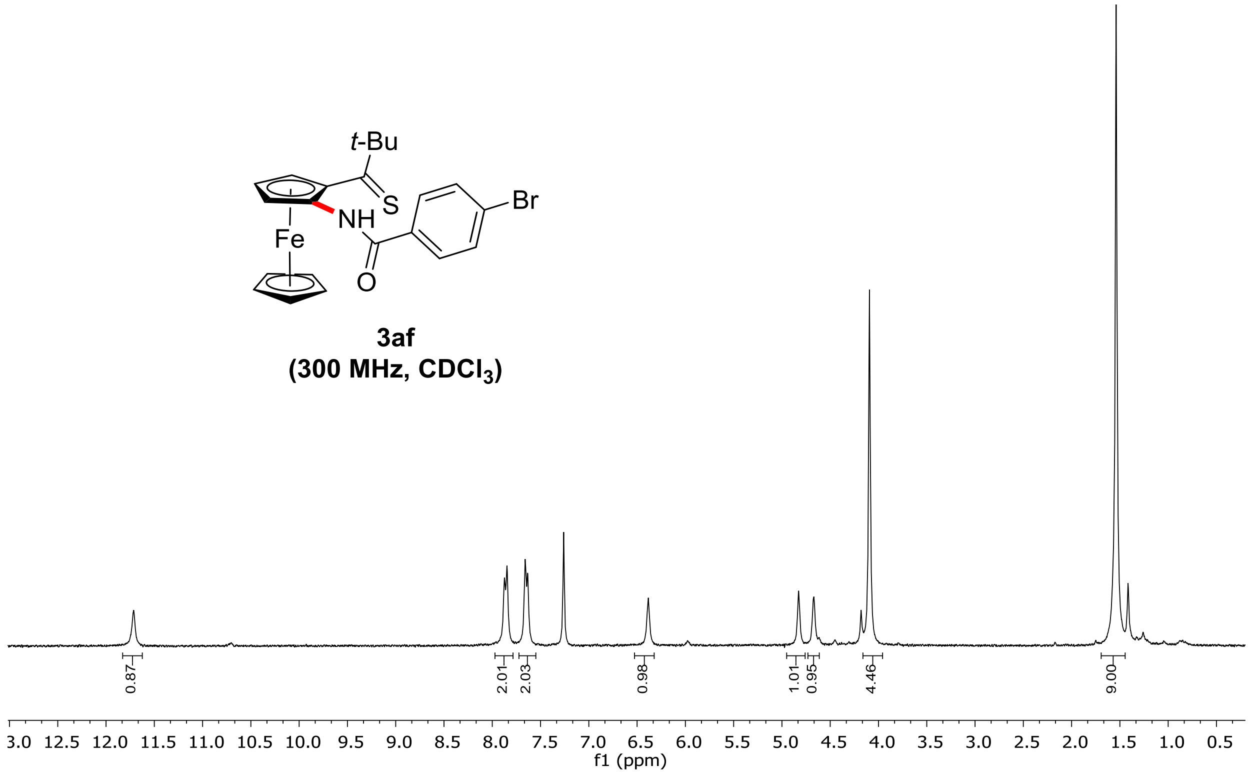
t-Bu
S
NH
Br
Fe
O
3af
(300 MHz, CDCl3)
0.87
2.01
2.03
0.98
1.01
0.95
4.46
9.00
3.0 12.5 12.0 11.5 11.0 10.5 10.0 9.5 9.0 8.5 8.0 7.5 7.0 6.5 6.0 5.5 5.0 4.5 4.0 3.5 3.0 2.5 2.0 1.5 1.0 0.5
f1 (ppm)

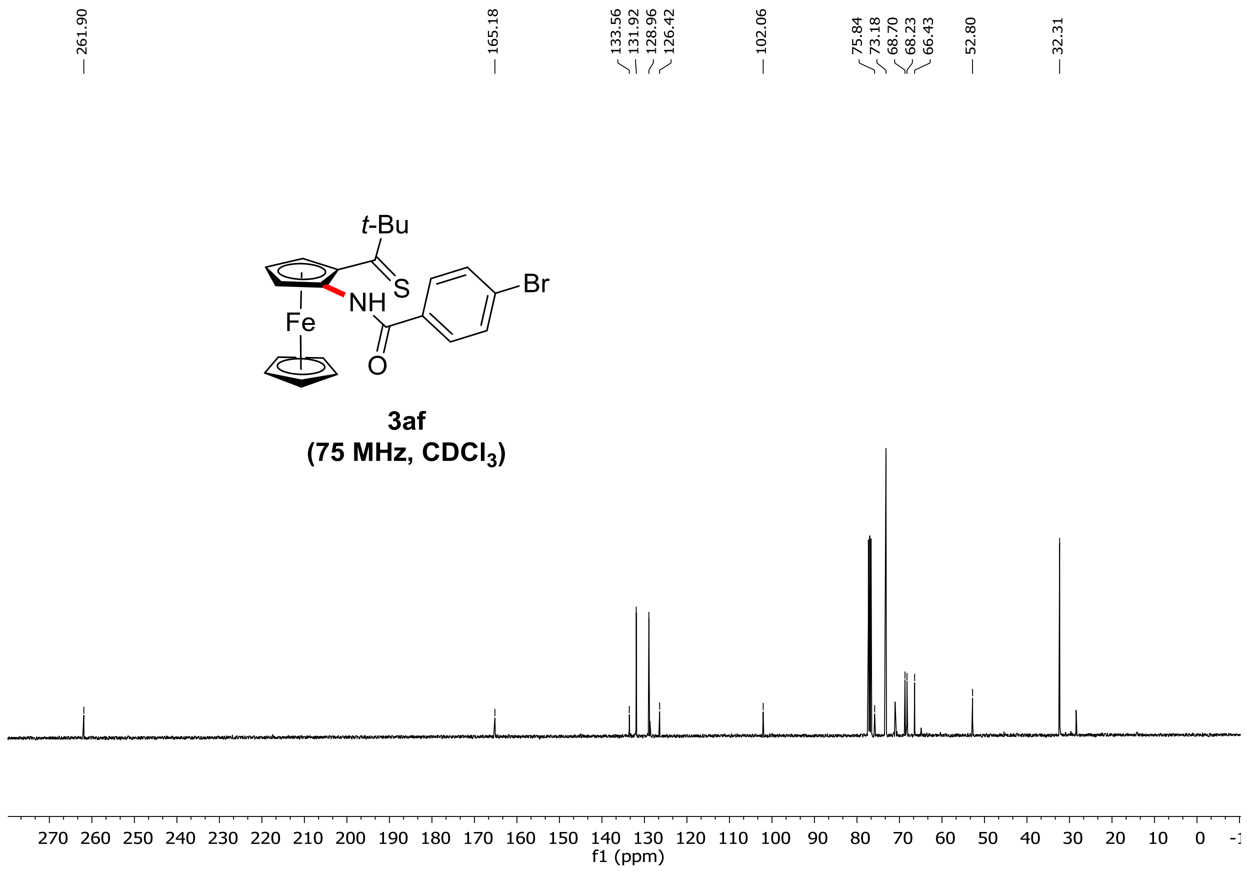

3af
(75 MHz, $CDCl_3$)

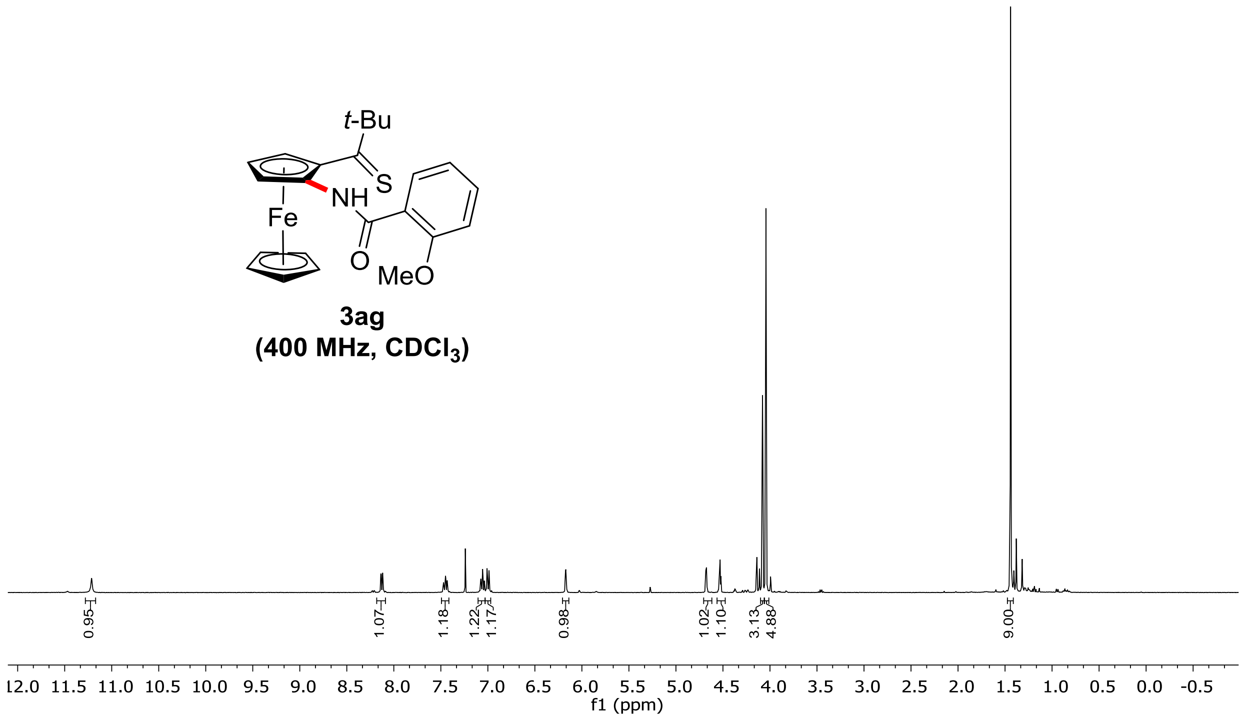

3ag
(400 MHz, $CDCl_3$)

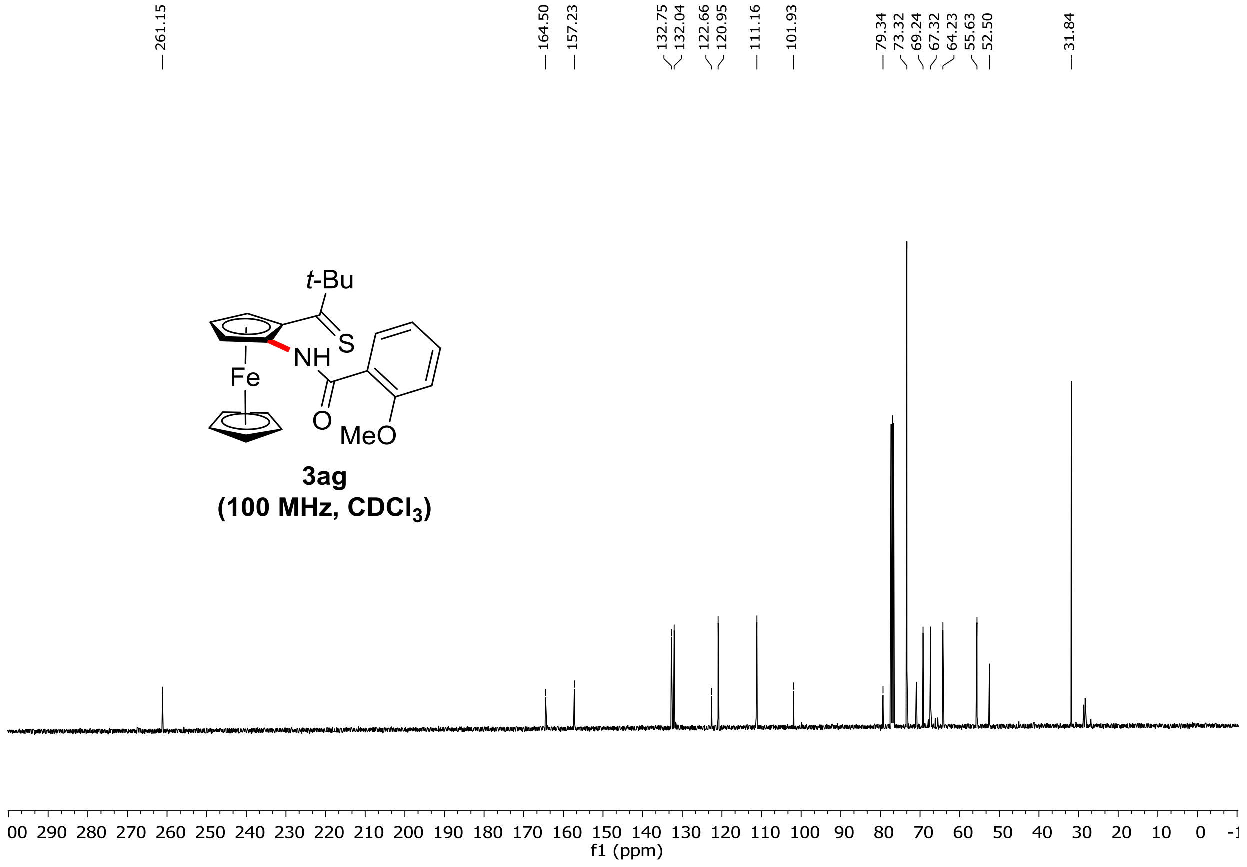
261.15
164.50
157.23
132.75
132.04
122.66
120.95
111.16
101.93
79.34
73.32
69.24
67.32
64.23
55.63
52.50
31.84
t-Bu
S
NH
Fe
O
MeO
3ag
(100 MHz, CDCl3)
f1 (ppm)

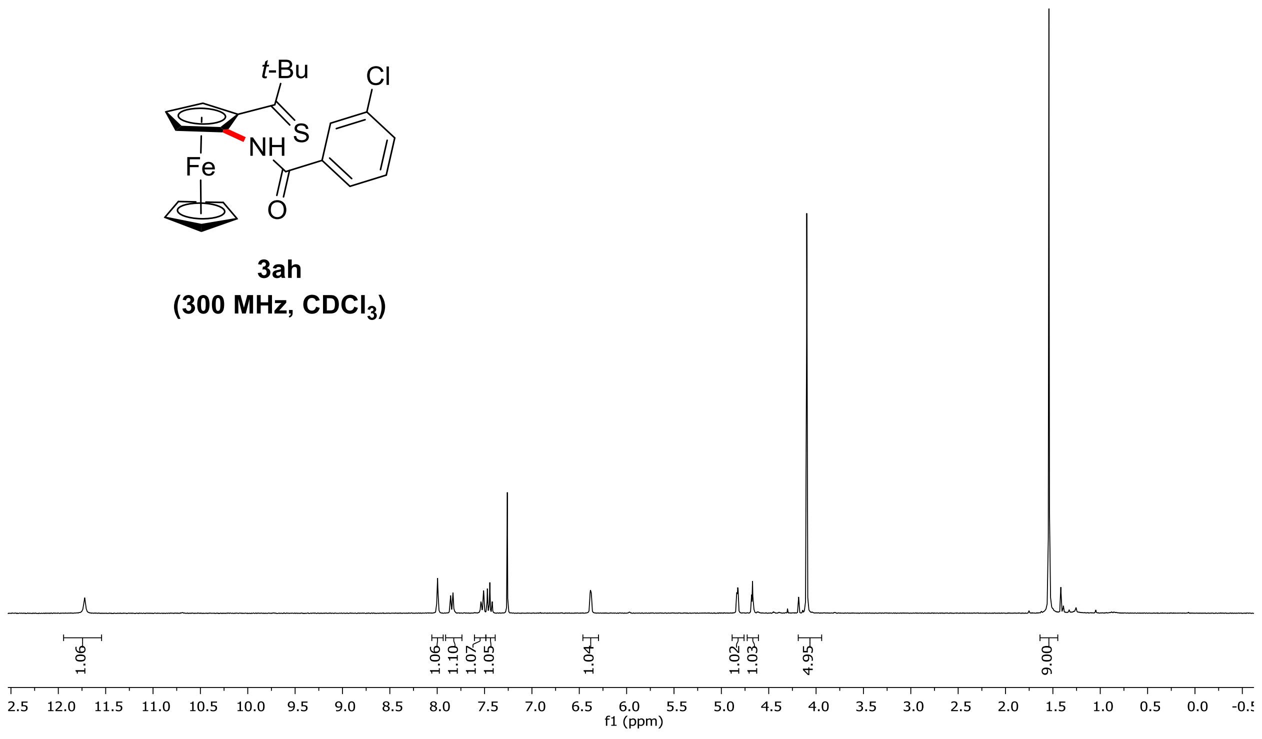
t-Bu
Cl
S
NH
Fe
O
3ah
(300 MHz, CDCl3)
1.06
1.06
1.10
1.07
1.05
1.04
1.02
1.03
4.95
9.00
f1 (ppm)

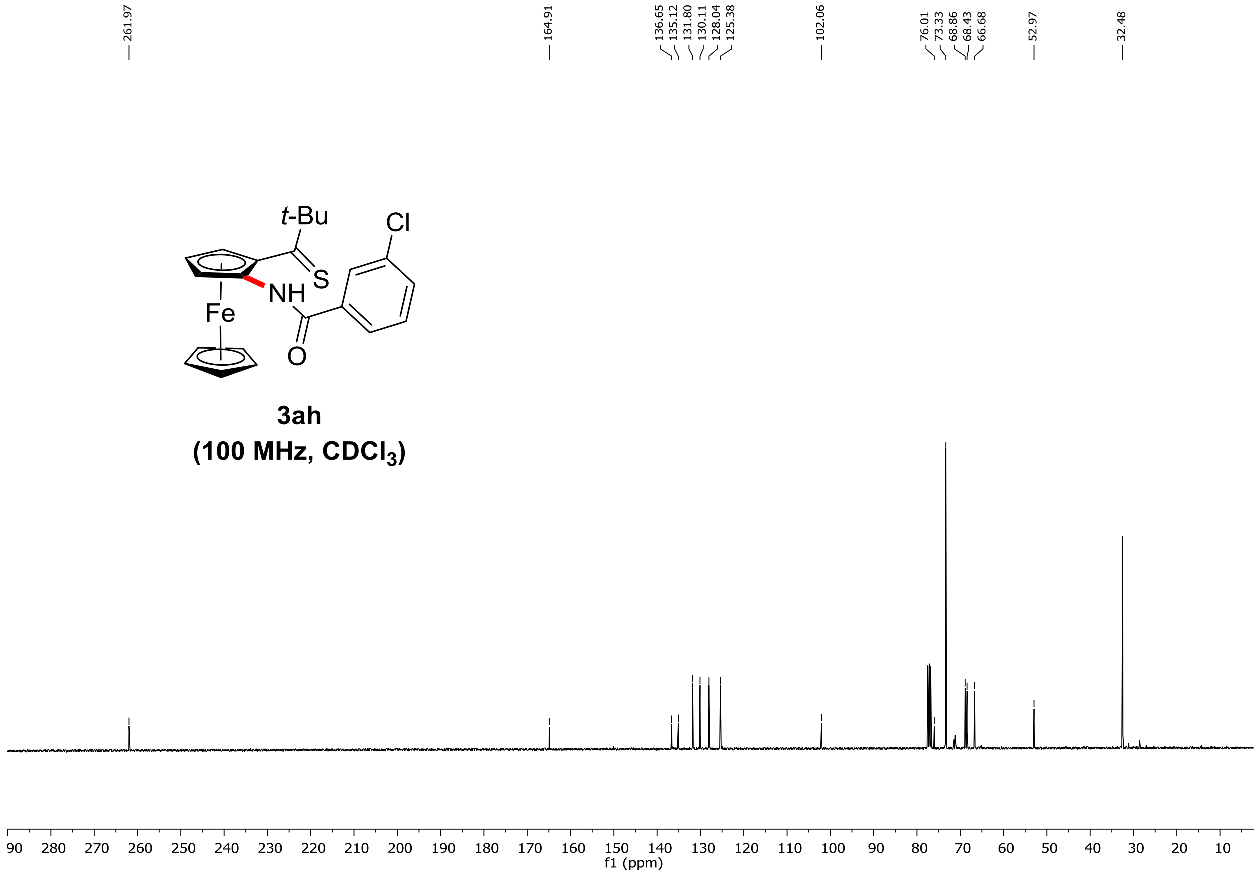

3ah
(100 MHz, $CDCl_3$)

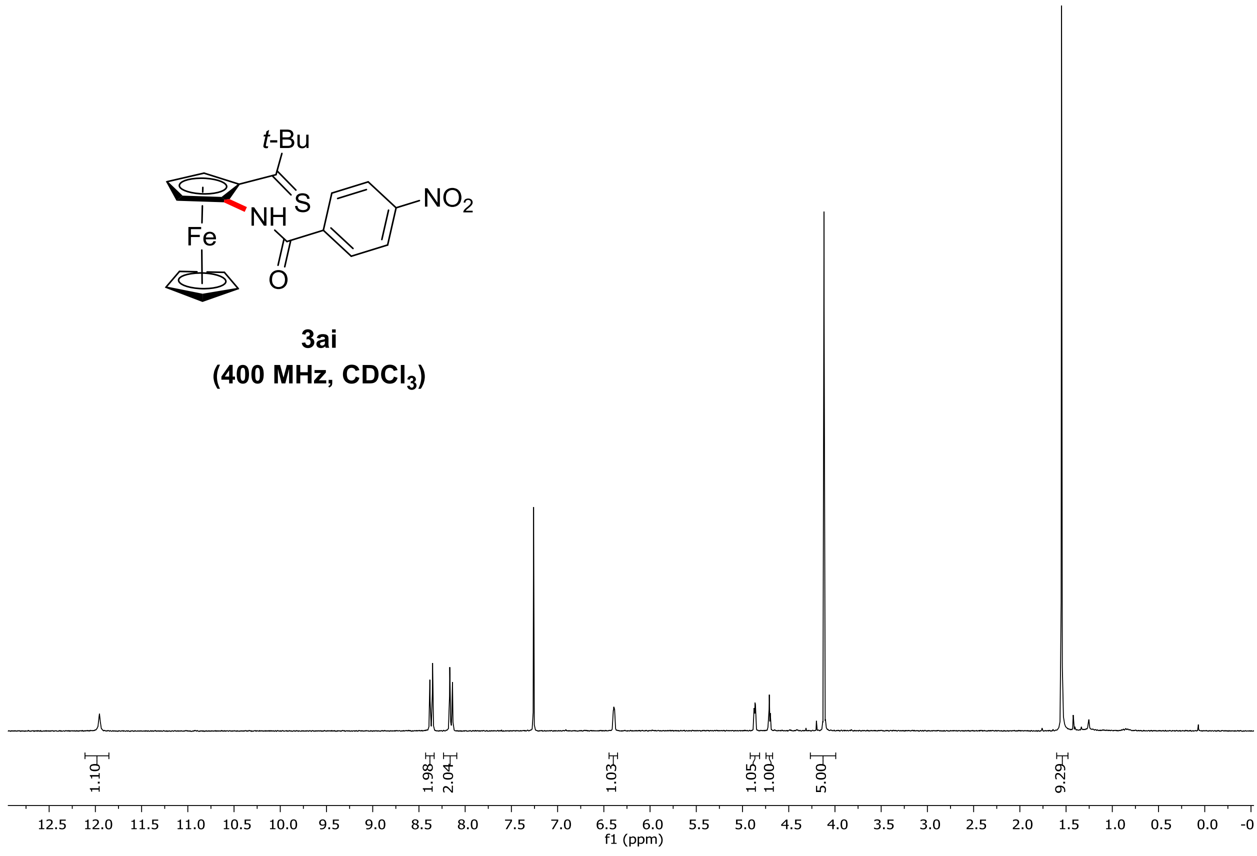

3ai
(400 MHz, $CDCl_3$)

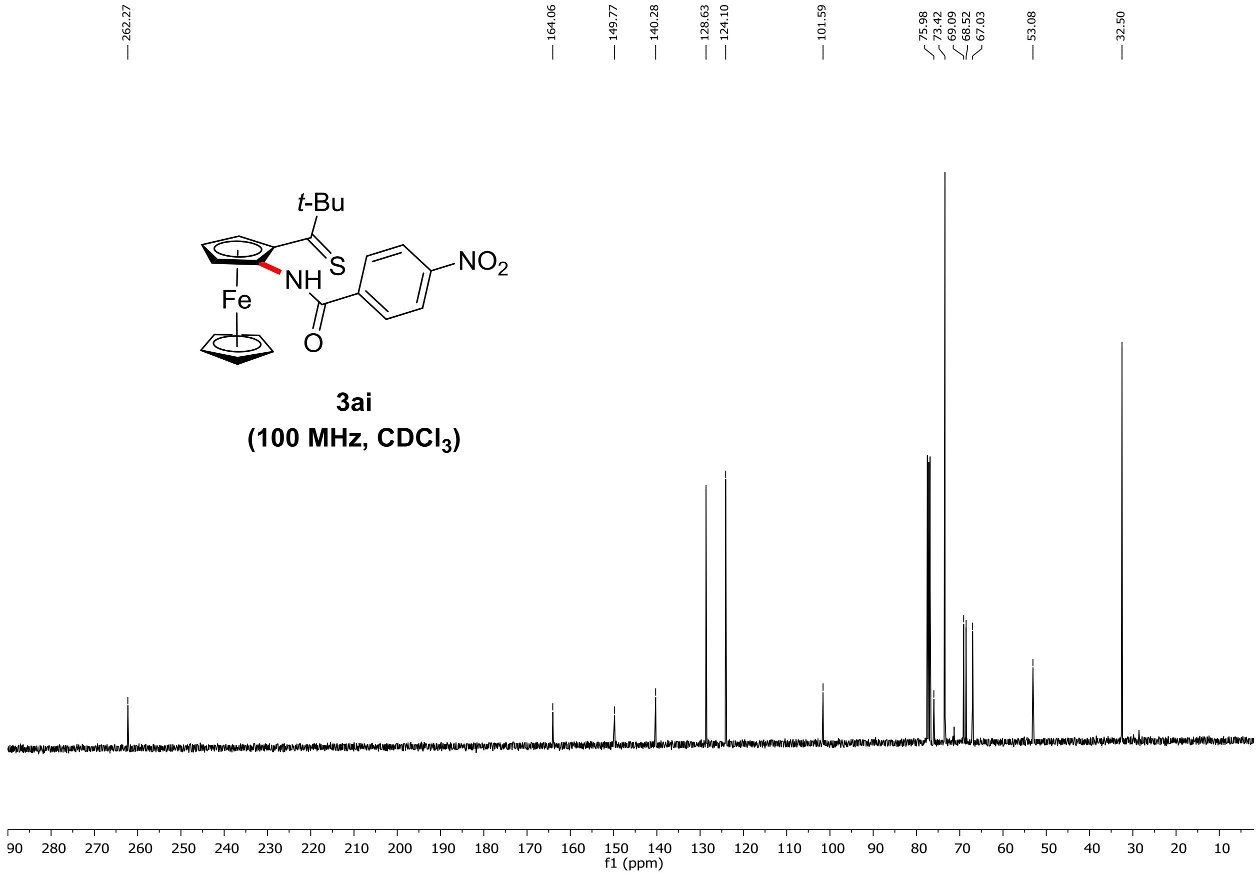
262.27
164.06
149.77
140.28
128.63
124.10
101.59
75.98
73.42
69.09
68.52
67.03
53.08
32.50
t-Bu
S
NH
NO2
Fe
O
3ai
(100 MHz, CDCl3)
f1 (ppm)

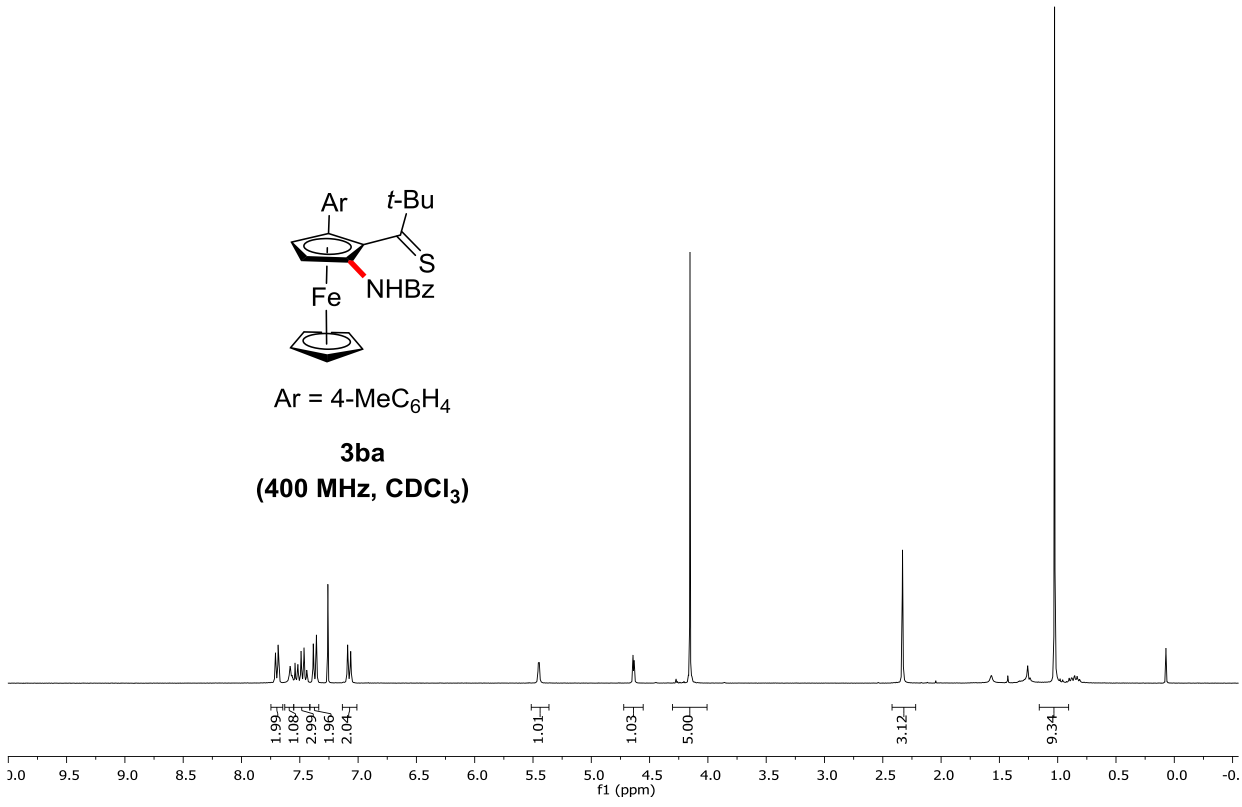
Ar
t-Bu
S
NHBz
Fe
Ar = 4-MeC6H4
3ba
(400 MHz, CDCl3)
1.99
1.08
2.99
1.96
2.04
1.01
1.03
5.00
3.12
9.34
f1 (ppm)

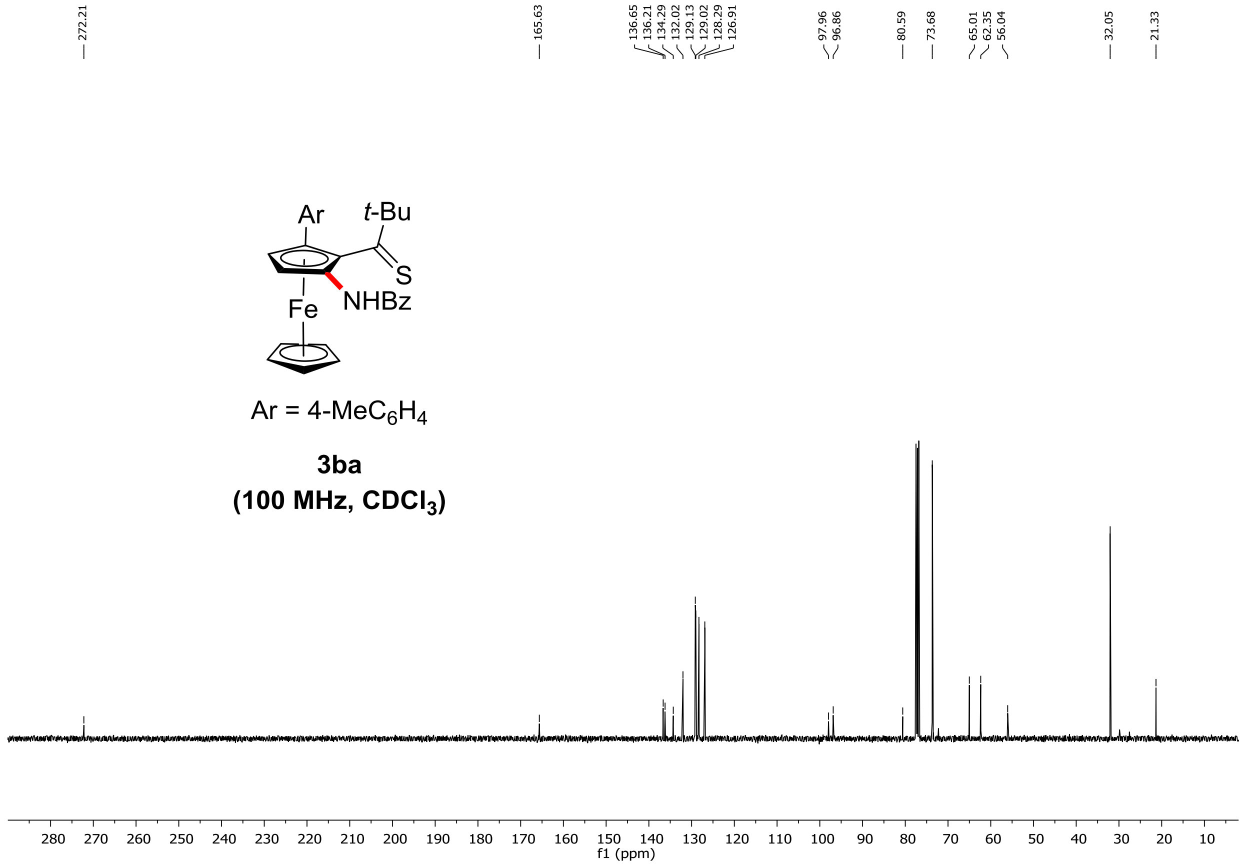

Ar = 4-MeC_6H_4

3ba
(100 MHz, $CDCl_3$)

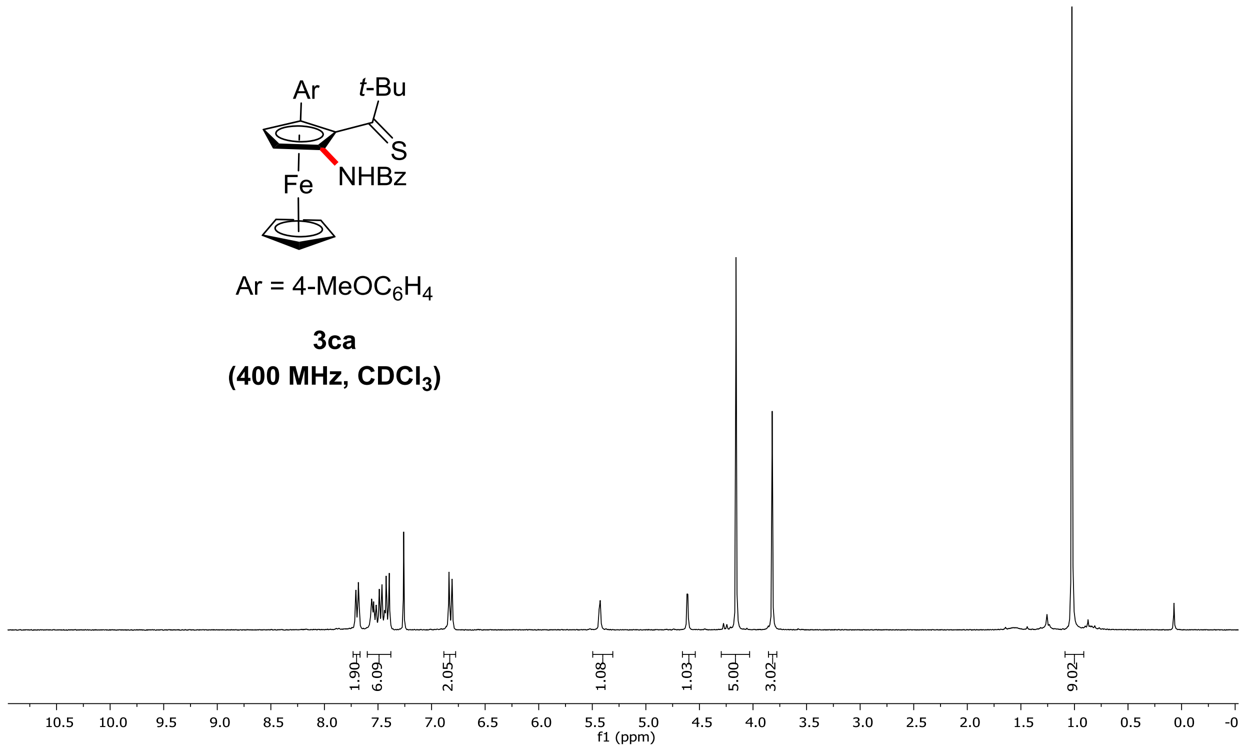

Ar = 4-$MeOC_6H_4$

3ca
(400 MHz, $CDCl_3$)

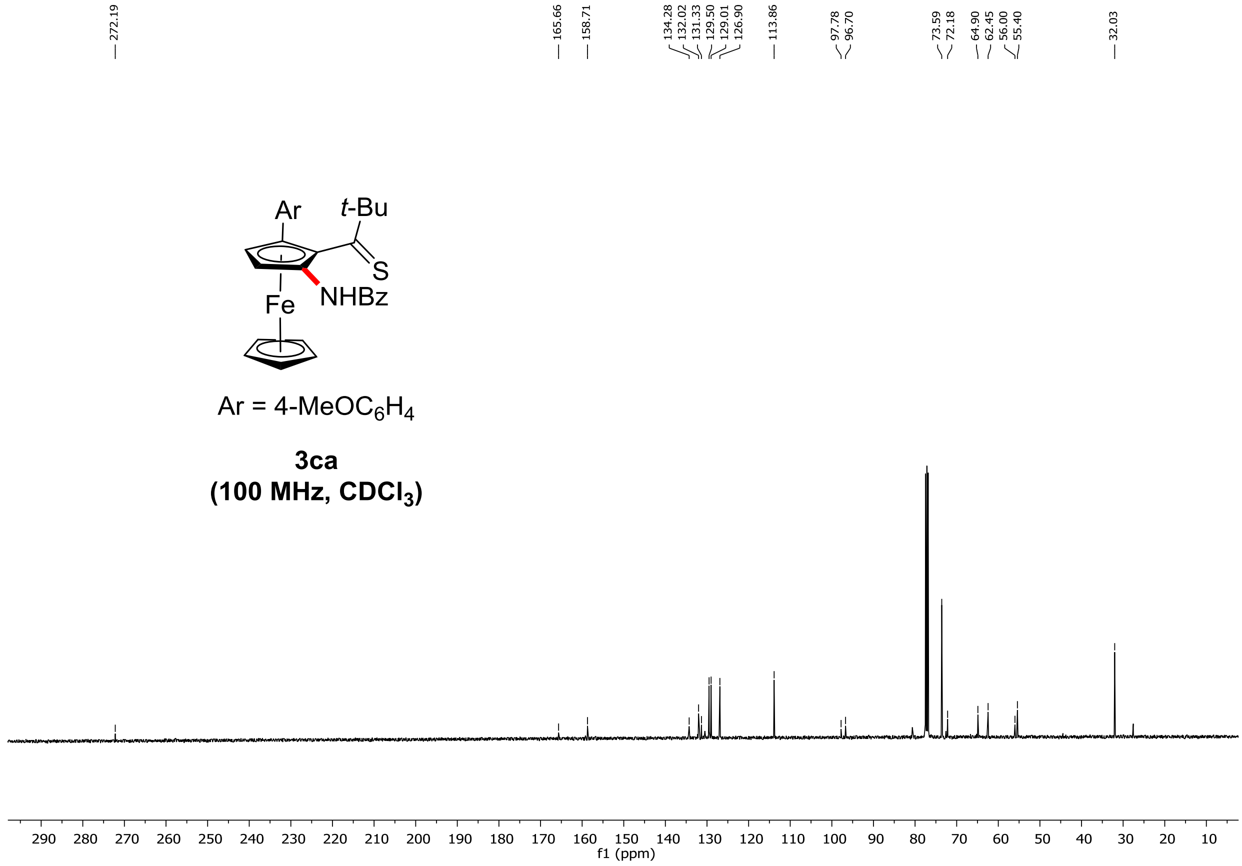
272.19
165.66
158.71
134.28
132.02
131.33
129.50
129.01
126.90
113.86
97.78
96.70
73.59
72.18
64.90
62.45
56.00
55.40
32.03
Ar
t-Bu
S
Fe
NHBz
Ar = 4-MeOC6H4
3ca
(100 MHz, CDCl3)
290 280 270 260 250 240 230 220 210 200 190 180 170 160 150 140 130 120 110 100 90 80 70 60 50 40 30 20 10
f1 (ppm)

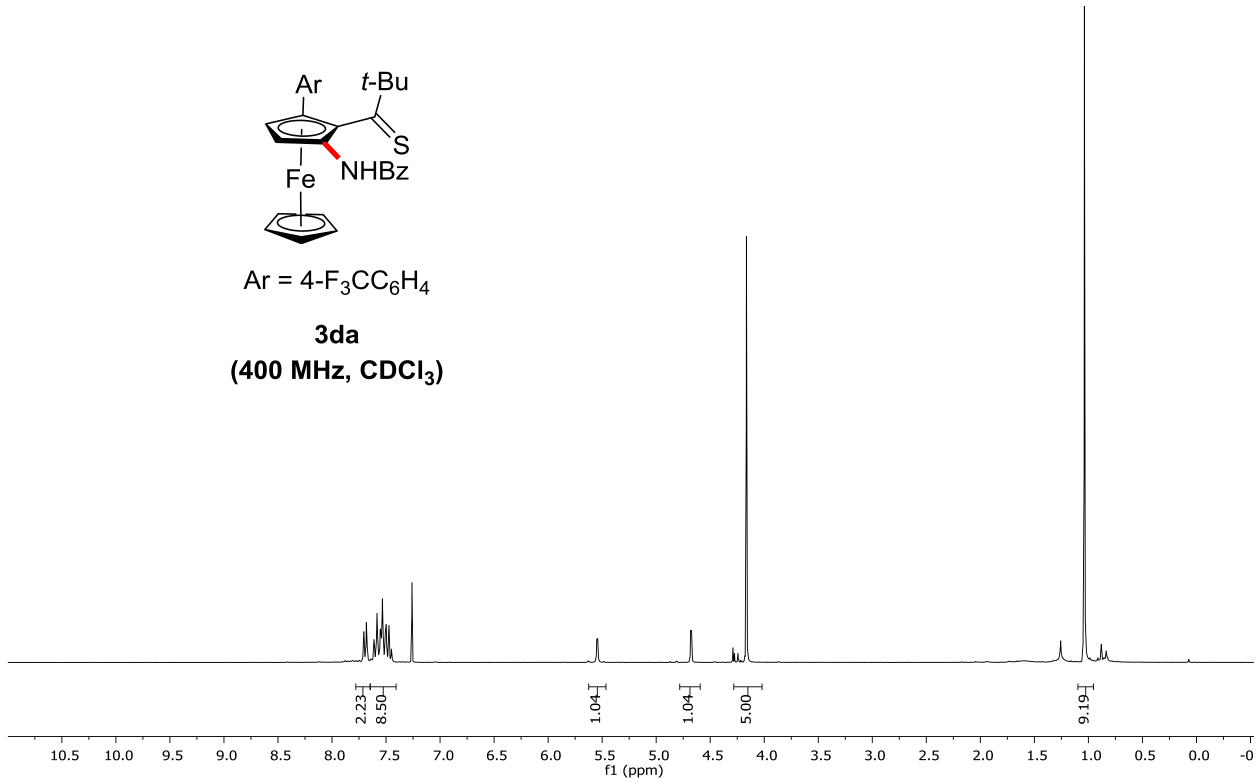
Ar
t-Bu
S
Fe
NHBz
Ar = 4-F3CC6H4
3da
(400 MHz, CDCl3)
2.23
8.50
1.04
1.04
5.00
9.19
10.5 10.0 9.5 9.0 8.5 8.0 7.5 7.0 6.5 6.0 5.5 5.0 4.5 4.0 3.5 3.0 2.5 2.0 1.5 1.0 0.5 0.0 -0
f1 (ppm)

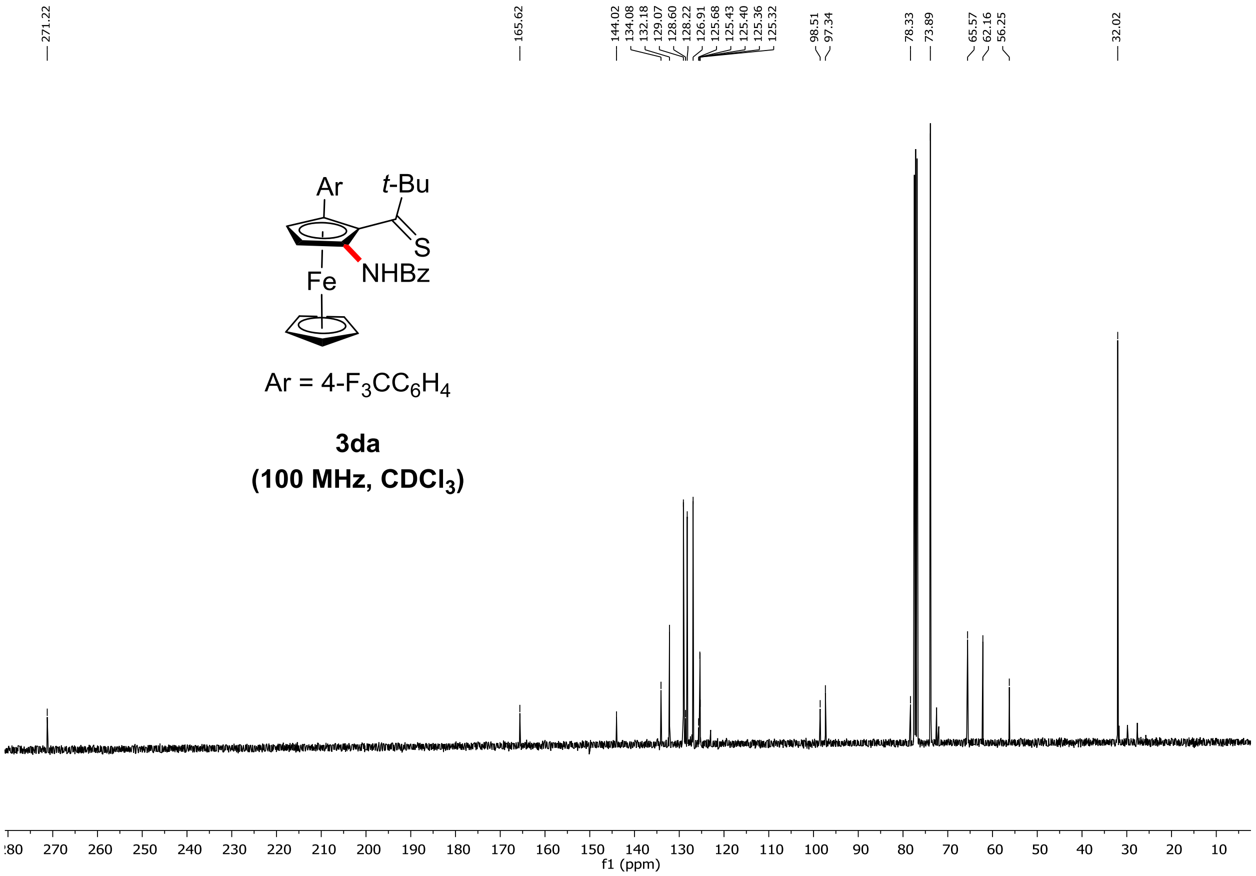
271.22
165.62
144.02
134.08
132.18
129.07
128.60
128.22
126.91
125.68
125.43
125.40
125.36
125.32
98.51
97.34
78.33
73.89
65.57
62.16
56.25
32.02
Ar
t-Bu
S
Fe
NHBz
Ar = 4-F$_3$CC$_6$H$_4$
3da
(100 MHz, CDCl$_3$)
f1 (ppm)

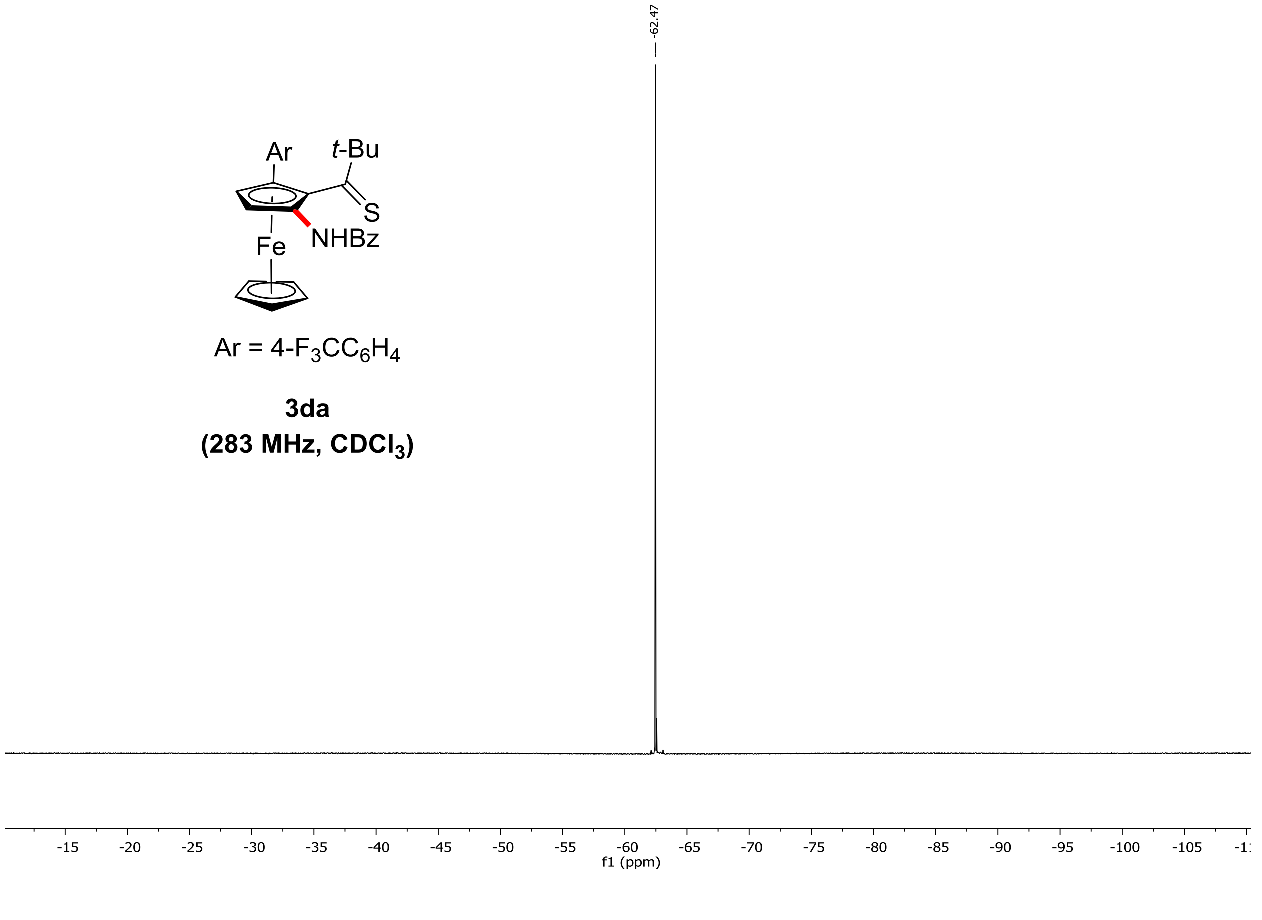
-62.47
Ar
t-Bu
S
Fe
NHBz
Ar = 4-F$_3$CC$_6$H$_4$
3da
(283 MHz, CDCl$_3$)
f1 (ppm)

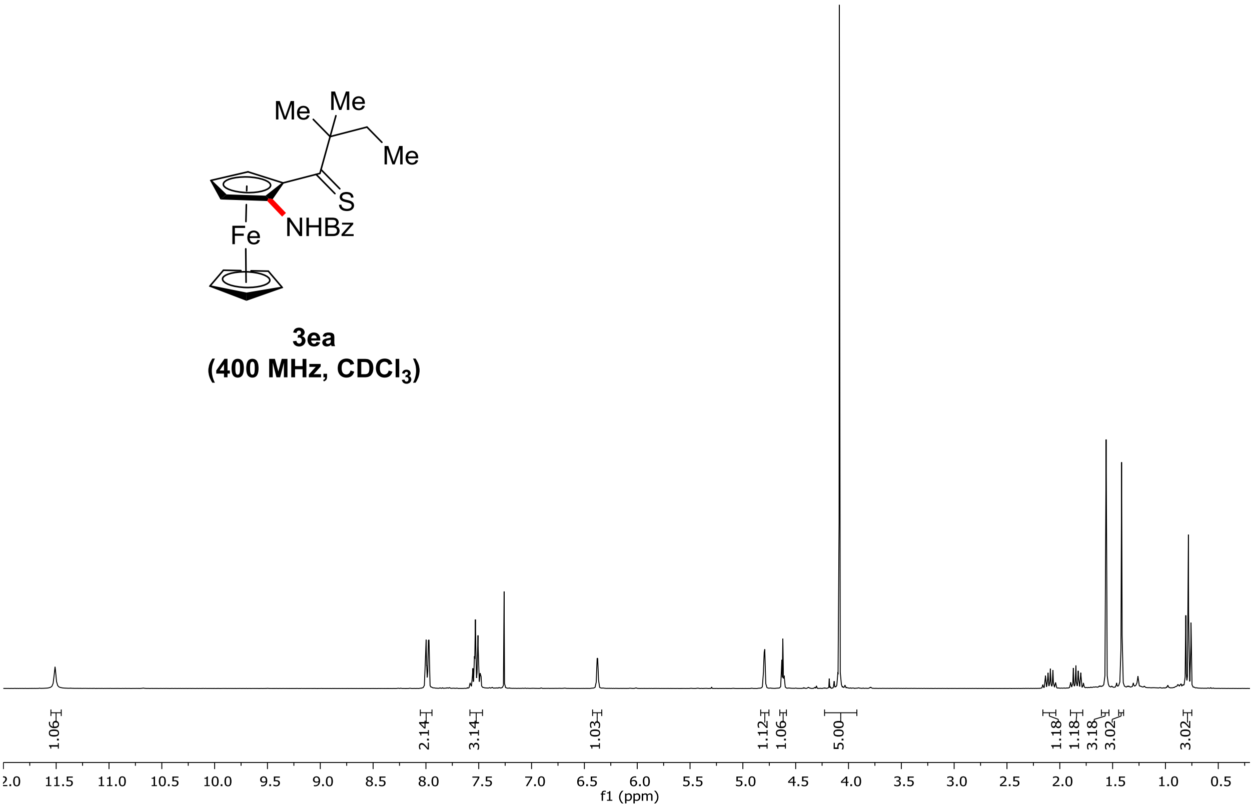

Me
Me
Me
S
Fe
NHBz
3ea
(400 MHz, CDCl3)
1.06
2.14
3.14
1.03
1.12
1.06
5.00
1.18
1.18
3.18
3.02
3.02
f1 (ppm)

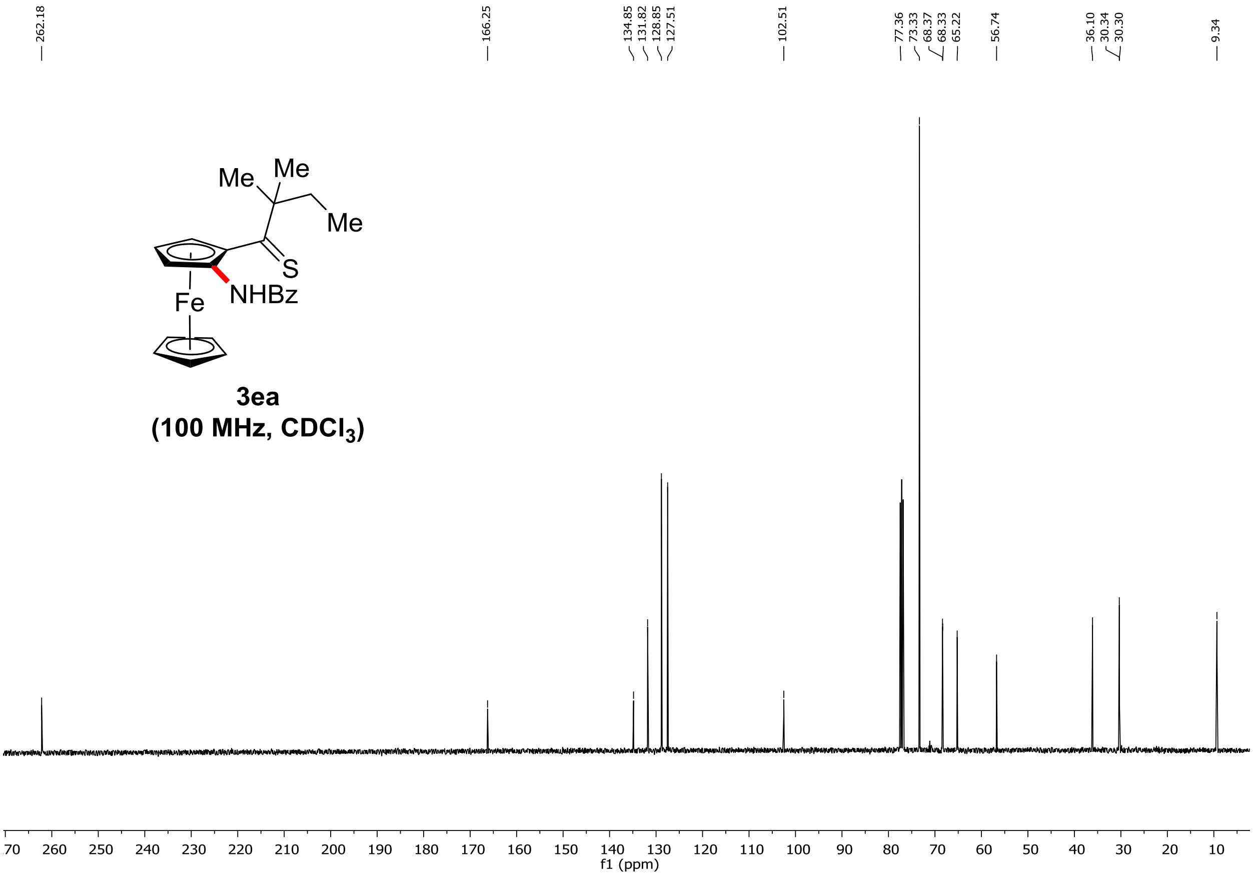

262.18
166.25
134.85
131.82
128.85
127.51
102.51
77.36
73.33
68.37
68.33
65.22
56.74
36.10
30.34
30.30
9.34
Me
Me
Me
S
Fe
NHBz
3ea
(100 MHz, CDCl3)
f1 (ppm)

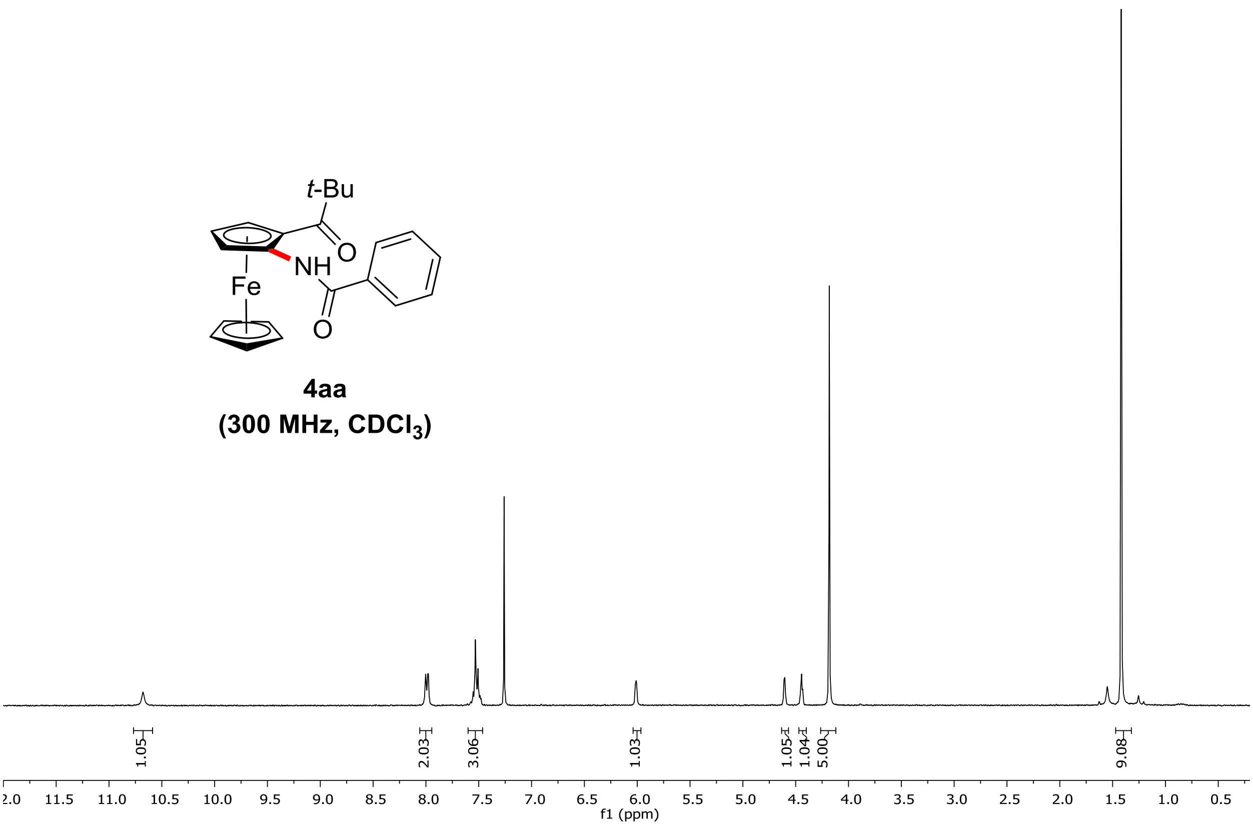

4aa
(300 MHz, $CDCl_3$)

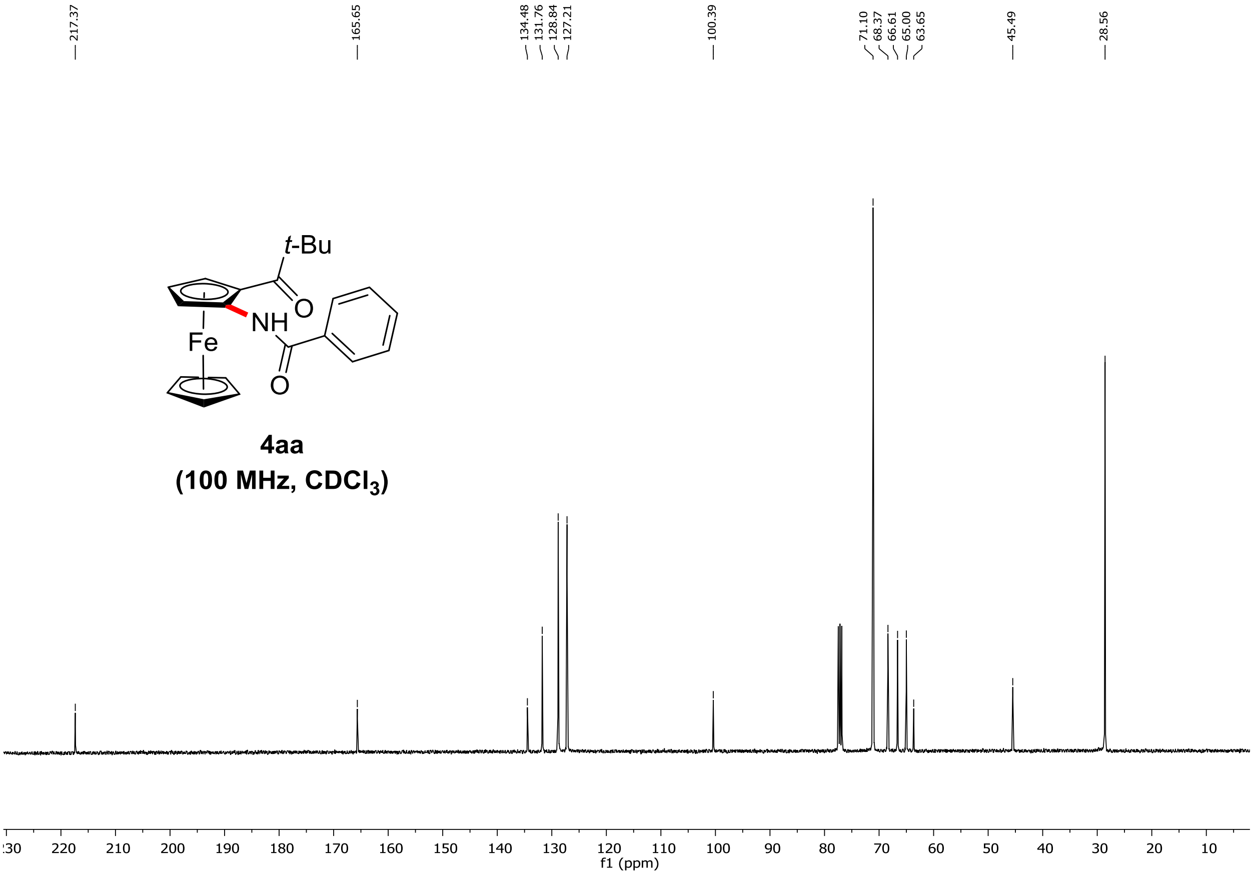

4aa
(100 MHz, $CDCl_3$)
